# Mn(III)-Initiated Facile Oxygenation of Heterocyclic 1,3-Dicarbonyl Compounds

**DOI:** 10.3390/molecules16119562

**Published:** 2011-11-16

**Authors:** Md. Taifur Rahman, Md. Aminul Haque, Hikaru Igarashi, Hiroshi Nishino

**Affiliations:** Department of Chemistry, Graduate School of Science and Technology, Kumamoto University, Kurokami 2-39-1, Kumamoto 860-8555, Japan

**Keywords:** hydroperoxides, peroxy radicals, aerobic oxidation, redox, 2-hydroxy-1,3-dicarbonyl compounds

## Abstract

The Mn(III)-initiated aerobic oxidation of heterocyclic 1,3-dicarbonyl compounds, such as 4-alkyl-1,2-diphenylpyrazolidine-3,5-diones, 1,3-dialkylpyrrolidine-2,4-diones, 3-alkyl-1,5-dimethylbarbituric acids, and 3-butyl-4-hydroxy-2-quinolinone gave excellent to good yields of the corresponding hydroperoxides, which were gradually degraded by exposure to the metal initiator after the reaction to afford the corresponding alcohols. The synthesis of 30 heterocyclic 1,3-dicarbonyl compounds, the corresponding hydroperoxides and the 10 alcohols, their characterization, and the limitations of the procedure are described. In addition, the mechanism of the hydroperoxidation and the redox decomposition of the hydroperoxides are discussed.

## 1. Introduction

Oxygenation of enolates has been considered as an important organic transformation [1,2,3], because the *α*-hydroxycarbonyl moiety is used as a starting material or an intermediate for the synthesis of some natural products [4,5,6,7,8]. In particular, the 2-hydroxy-1,3-dicarbonyl moiety can be found in biologically important compounds such as indole alkaloids, e.g., vindorosine and vindoline [9,10,11,12], the cyclopentenoid kjellamanianone [13], and tetracycline-type antibiotics such as doxycycline [14,15]. 4-Butyl-1,2-diphenylpyrazolidine-3,5-dione (phenylbutazone), a nonstereoidal drug, is an efficient reducing cofactor for the peroxidase activity of prostaglandin H synthase [16,17]. Phenylbutazone inhibits the production of lipid mediators causing inflammation but paradoxically performs this *via* the intermediacy of the peroxy radical and hydroperoxide, which may themselves be proinflammatory. 4-Hydroperoxyphenylbutazone shows a significantly stronger cardiodepressive and coronary constricting effects compared to phenylbutazone itself, 4-hydroxyphenylbutazone, and the ring-opened decomposition product of the hydroperoxide [18]. These phenomena could shed light on the significance of the 4-hydroperoxyphenylbutazone regarding the antiinflammatory or other biological activities of phenylbutazone [19,20] and could explain the side effects such as gastric irritation and toxicity associated with phenylbutazone. Although many reagents have been utilized for the introduction of an oxygen functionality at the 2-position of 1,3-dicarbonyl compounds, the hydroxyl functionality could be introduced at the *α*-position in most of the cases [21,22,23,24,25,26,27,28,29,30].

**Scheme 1:**
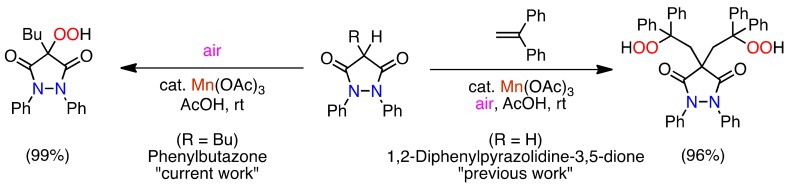
Mn(III)-assisted aerobic oxidation of 1,2-diphenylpyrazolidine-3,5-dione (R = H) [38,39] and phenylbutazone (R = Bu) [42].

In connection with our current investigation of the Mn(OAc)_3_-assisted aerobic oxidation [31,32,33,34,35,36], we found the double hydroperoxyalkylaton of barbituric acids [37], pyrazolidine-3,5-diones (Scheme 1) [38,39], 4-hydroxy-1*H*-quinolin-2-ones [40], and tetronic acid [41]. The reaction could not be stopped at the monohydroperoxyalkylation stage.

Surprisingly, when phenylbutazone was treated under similar reaction conditions in the absence of an alkene, direct hydroperoxidation occurred and the corresponding solid 4-hydroperoxy-phenylbutazone was produced quantitatively (Scheme 1) [42]. We foresaw that the reaction could be very useful for the synthesis of hydroperoxides under very mild aerobic oxidation conditions. Therefore, in order to examine the applicability of Mn(OAc)_3_-assisted *α*-hydroperoxidation, biologically important heterocyclic 1,3-dicarbonyl compounds, e.g., 1,3-dialkylpyrrolidine-2,4-diones **3**, 3-alkyl-1,5-dimethylbarbituric acids **5**, and 3-butyl-4-hydroxy-2-quinolinone (**7**) as well as various 4-monoalkyl-substituted pyrazolidine-3,5-diones **1** were subjected to the reaction, and we obtained very similar results, whereby the corresponding hydroperoxides were obtained in excellent to good yields. In this paper, the full results of the earlier work [42], applications and limitations of the Mn(III)-initiated direct *α*-hydroperoxidation, and the related reactions are described. Specifically, the full characterization of all the starting materials **1**, **5**, and **7**, and all the hydroperoxides published in the reference [42], the full results of the hydroperoxidation of 1,3-dialkylpyrrolidine-2,4-diones **3**, including the characterization of the corresponding hydroperoxides, and the mechanism of the Mn(III)-initiated autoxidation are discussed. In addition, the limitations of the hydroperoxidation are mentioned using the reaction of acyclic amides, cyclic ketones and esters as model, and the synthesis of 2-hydroxy-1,3-dicarbonyl compounds using the reduction of the hydroperoxides is described.

## 2. Results and Discussion

### 2.1. Aerobic Oxidation of 4-Alkyl-substituted Pyrazolidine-3,5-diones ***1a–h***

**Scheme 2:**
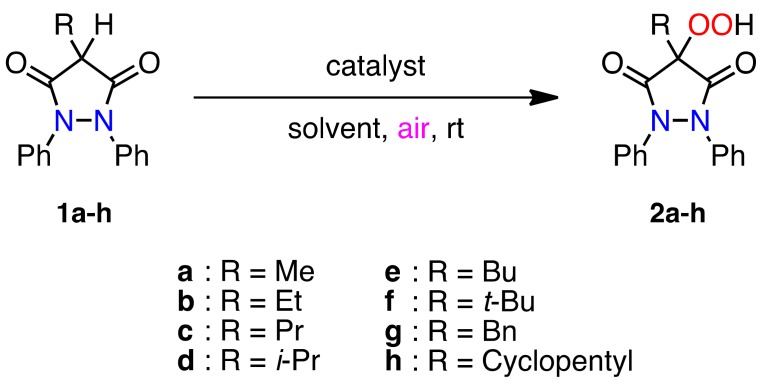
Autoxidation of 4-alkyl-substituted 1,2-diphenylpyrazolidine-3,5-diones **1a–h**.

4-Alkyl-1,2-diphenylpyrazolidine-3,5-diones **1a–h** were prepared by a simple condensation reaction between the corresponding alkyl malonate and 1,2-diphenylhydrazine in the presence of NaH in boiling chlorobenzene [17]. With the pyrazolidinediones **1a–h** in hand, a mixture of phenylbutazone (**1e**, 1 mmol) and Mn(OAc)_3_ (0.1 mmol) in glacial AcOH (30 mL) was stirred at 23 °C under an aerobic atmosphere, and the desired 4-hydroperoxypyrazolidinedione **2e** was produced in quantitative yield (Scheme 2 and Table 1, entry 9). To scrutinize the role of Mn(OAc)_3_ in the aerobic oxidation, we carried out some reactions of 4-*i*-propypyrazolidinedione **1d** under different reaction conditions. When the pyrazolidinedione **1d** was stirred in the absence of Mn(OAc)_3_, no conversion to the hydroperoxide **2d** was observed (entry 4). Using Cu(OAc)_2_ and ammonium cerium(IV) nitrate (CAN) as the catalyst was not effective in the autoxidation (entries 5 and 6). Therefore, it could be inferred that Mn(OAc)_3_ was essential for the hydroperoxidation and that the reaction is neither thermal nor it is induced by sunlight or visible light. While carrying out the reaction of **1d** in the presence or absence of light (entries 7 and 8), there is no alteration in the yield of the hydroperoxide **2d**. Thus, the reaction is not light-assisted. Various alkyl groups were used as the substituent at the 4-position of the pyrazolidinedione, but in all cases similar quantitative conversion to the corresponding hydroperoxides was observed. The results are summarized in Table 1.

**Table 1:** Aerobic oxidation of 4-alkyl-substituted pyrazolidinediones **1a–h** in the presence of a metal oxidant ^a^.

Entry	1	Oxidant	1:oxidant ^b^	Time/h	Product/yield (%) ^c^
1	**1a**	Mn(OAc)_3_	1:0.1	2	**2a** (95)
2	**1b**	Mn(OAc)_3_	1:0.1	2	**2b** (95)
3	**1c**	Mn(OAc)_3_	1:0.1	2	**2c** (99)
4	**1d**	none	-	3	Recovery of **1d** (100)
5	**1d**	Cu(OAc)_2_	1:0.2	15	Recovery of **1d** (94)
6 ^d^	**1d**	CAN ^e^	1:0.1	1	**2d** (40)
7	**1d**	Mn(OAc)_3_	1:0.1	2	**2d** (97)
8 ^f^	**1d**	Mn(OAc)_3_	1:0.1	2	**2d** (96)
9	**1e**	Mn(OAc)_3_	1:0.1	2	**2e** (99)
10	**1f**	Mn(OAc)_3_	1:0.1	10 min	**2f** (95)
11	**1g**	Mn(OAc)_3_	1:0.1	2	**2g** (97)
12	**1h**	Mn(OAc)_3_	1:0.1	2	**2h** (99)

^a^ The reaction of **1** (1 mmol) was carried out in AcOH at ambient temperature in air; ^b^ Molar ratio; ^c^ Isolated yield is based on the pyrazolidinedione **1** used; ^d^ The reaction was carried out in MeOH at 0 °C; ^e^ Ammonium cerium(IV) nitrate; ^f^ The reaction was carried out in the dark.

### 2.2. Aerobic Oxidation of 3-Alkyl-substituted Pyrrolidine-2,4-diones ***3a–p***

**Scheme 3:**
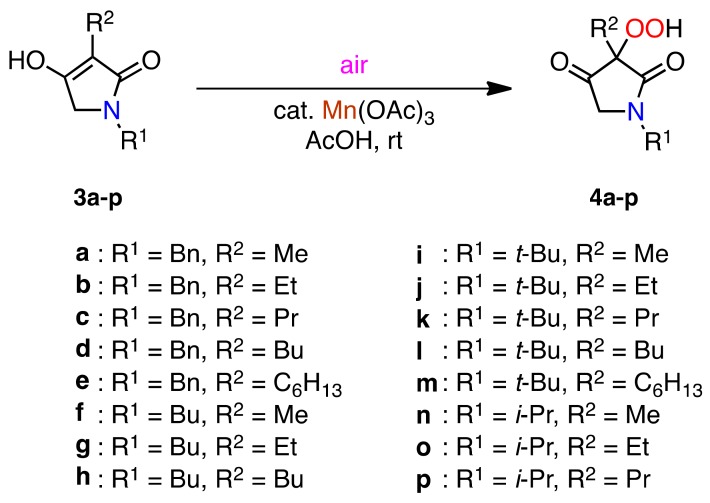
Aerobic oxidation of 1,3-dialkyl-substituted pyrrolidine-2,4-diones **3a–p**.

We were pleased to confirm that the specific hydroperoxidation succeeded using pyrazolidinediones **1a–h**, so we next applied the reaction to 1,3-dialkyl-substituted pyrrolidine-2,4-diones **3a–p** which are structurally similar to the pyrazolidinediones. The pyrrolidine-2,4-diones **3a–p** were prepared by the Dieckmann condensation of *N*-alkanoyl-*N*-alkylglycinates which were produced by the reaction of *α*-bromoacetate with alkylamines followed by alkanoylation with the corresponding alkanoyl chloride (see Experimental section) [43,44,45,46,47]. The 2,4-pyrrolidinediones **3a–p** exist as an enol form (3-alkyl-4-hydroxy-3-pyrrolin-2-ones) in an aprotic polar solvent, such as DMSO-*d*_6_ [48]. With the 2,4-pyrrolidinediones **3a–p** in hand, we first explored the hydroperoxidation of **3a**. When the reaction was carried out using a stoichiometric amount of Mn(OAc)_3_, the oxidant was consumed after 2 h, and the desired hydroperoxide **4a** was obtained in 65% yield (Scheme 3 and Table 2, entry 1).

In order to optimize the hydroperoxidation, the reaction was conducted under various reaction conditions, and we finally obtained the hydroperoxide **4a** in 94% yield under the conditions of room temperature reaction in air for 2 h using a catalytic amount of Mn(OAc)_3_ (entry 3). To expand the scope of the reaction, the reaction of other 2,4-pyrrolidinediones **3b–p** was conducted under the optimized reaction conditions and similar hydroperoxides **4b–p** were obtained in excellent yields (entries 4–18). However, the reactions using 1-ethyl-3-methylpyrrolidine-2,4-dione (**3q**) and ethyl 1-benzylpyrrolidine-2,4-dione-3-carboxylate (**3r**) gave intractable mixtures and the corresponding hydroperoxides were not isolated. In addition, the reaction of 1-benzyl-3-phenylpyrrolidine-2,4-dione (**3s**) failed because of the solubility problem. Although the 4-hydroperoxypyrazolidinediones **2a–h** were stable at ambient temperature in air, the 3-hydroperoxypyrrolidinediones **4a–p** gradually decomposed within 2 or 3 days and even when stored in a refrigerator at −20 °C for a half year.

**Table 2:** Aerobic oxidation of 1,3-dialkyl-substituted pyrrolidinediones **3a–p** ^a^.

Entry	3	3:Mn(OAc)_3_^b^	Time/h	Product/yield (%) ^c^
1	**3a**	1:1	2	**4a** (65)
2	**3a**	1:0.1	14	**4a** (68)
3	**3a**	1:0.1	2	**4a** (94)
4	**3b**	1:0.1	2	**4b** (95)
5	**3c**	1:0.1	2	**4c** (98)
6	**3d**	1:0.1	2	**4d** (93)
7	**3e**	1:0.1	2	**4e** (96)
8	**3f**	1:0.1	1.5	**4f** (91)
9	**3g**	1:0.1	1.5	**4g** (90)
10	**3h**	1:0.1	1.5	**4h** (95)
11	**3i**	1:0.1	1.5	**4i** (90)
12	**3j**	1:0.1	1.5	**4j** (96)
13	**3k**	1:0.1	1.5	**4k** (99)
14	**3l**	1:0.1	1.5	**4l** (99)
15	**3m**	1:0.1	1.5	**4m** (98)
16	**3n**	1:0.1	1.5	**4n** (95)
17	**3o**	1:0.1	1.5	**4o** (97)
18	**3p**	1:0.1	1.5	**4p** (98)

^a^ The reaction of **3** (1 mmol) was carried out in AcOH (25 mL) at ambient temperature in air; ^b^ Molar ratio; ^c^ The yield is based on the amount of **3** used.

### 2.3. Aerobic Oxidation of 3-Alkyl-1,5-dimethylbarbituric Acids ***5a–e*** and 3-Butyl-4-hydroxy-2-quinolinone *(**7**)*

**Scheme 4:**
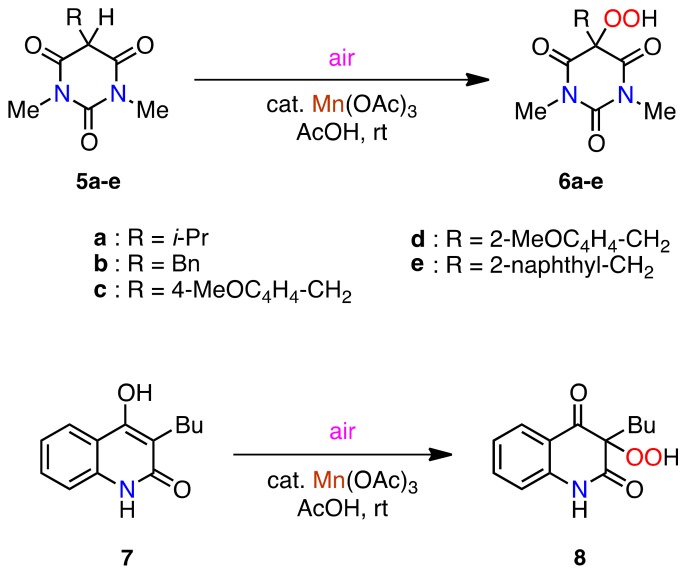
Aerobic oxidation of barbituric acids **5a–e** and 4-hydroxy-2-quinolinone **7**.

We next investigated the reaction of other biologically important heterocyclic 1,3-dicarbonyl compounds, such as 5-monosubstituted barbituric acids **5a–e** and 3-butyl-4-hydroxy-2-quinolinone **7**. The barbituric acids **5a–e** were prepared by Pd-catalyzed reductive alkylation of 1,3-dimethylbarbituric acid with acetone and aldehydes [49]. The quinolinone **7** was prepared by condensation of aniline and diethyl butylmalonate followed by dehydration (see Experimental section) [50,51,52]. With the barbituric acids **5a–e** and the quinolinone **7** in hand, the aerobic oxidation was carried out under similar reaction conditions, and very similar results were obtained, giving the corresponding hydroperoxides **6a–e** and **8**, respectively, in excellent to good yields (Scheme 4 and Table 3).

**Table 3:** Aerobic oxidation of barbituric acids **5a–e** and 4-hydroxy-2-quinolinone **7** ^a^.

Entry	Amide	Amide:Mn(OAc)_3_^b^	Time/h	Product/yield (%) ^c^
1	**5a**	1:0.1	4	**6a** (80)
2	**5b**	1:0.1	2	**6b** (90)
3	**5c**	1:0.1	4	**6c** (94)
4	**5d**	1:0.1	4	**6d** (94)
5	**5e**	1:0.1	2	**6e** (88)
6	**7**	1:0.1	2	**8** (57)

^a^ The reaction of **5** (1 mmol) was carried out in AcOH (30 mL) at ambient temperature in air; ^b^ Molar ratio; ^c^ The yield is based on the amount of **5** or **7** used.

### 2.4. Mechanism for the Formation of Hydroperoxides ***2***, ***4***, ***6***, and ***8***

**Scheme 5:**
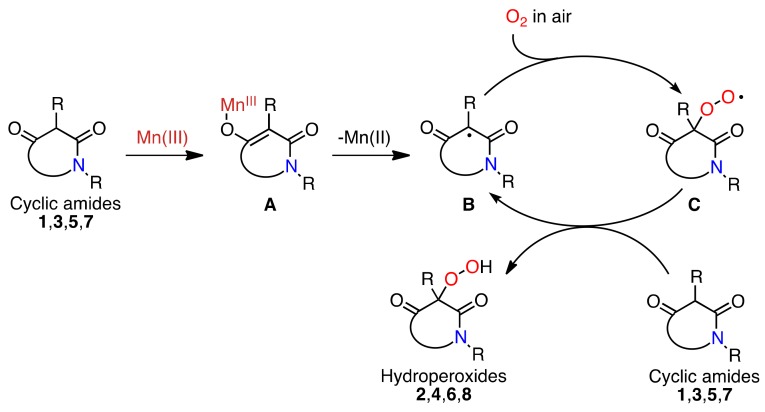
Mn(III)-initiated autoxidation of cyclic amides.

The aerobic oxidation might be explained by a radical chain mechanism. To rationalize our experimental results, we presume the formation of Mn(III)-enolate complex **A** *in situ* undergoing single-electron transfer (SET) to give 1,3-dicarbonyl radical **B** and the reduced Mn(II) (Scheme 5) [31,32,33,34,35,36]. This is the initiation step of the radical chain reaction. The 1,3-dicarbonyl radical **B** could be trapped by dissolved molecular oxygen in solution to produce the peroxy radical **C** [53,54]. The radical **C** could simply abstract a hydrogen atom from the cyclic amides to give the product hydroperoxides and another molecule of 1,3-dicarbonyl radical **B**, which continues the radical chain reaction. Since the redox potential (*E*°) of Mn(III)/Mn(II) is 1.54 V, it seems that the Mn(III) acts as an initiator rather than as a catalyst such as Cu(II)/Cu(I) (*E*° = 0.123 V) [55,56,57,58,59,60,61,62,63,64,65,66,67].

Involvement of the peroxy radical as well as the hydroperoxide intermediate in the course of transition metal-catalyzed autoxidation with different 1,3-dicarbonyl compounds has been proposed earlier [58,59]; however, there is only one report on the detection and identification of such a peroxy radical by electron spin resonance (ESR) [60]. To the best of our knowledge, there are no reports on the isolation and characterization of 2-hydroperoxy-1,3-dicarbonyl compounds in the transition metal-mediated autoxidation of the 1,3-dicarbonyl compounds, except for Hasegawa’s work [61]. They showed that introduction of the hydroperoxy functionality at the active methylene position of 1,3-dicarbonyl compounds could be accomplished by dye-sensitized photoreaction of singlet oxygen with the enolic 1,3-dicarbonyl compounds [61]. Although CAN also mediated the hydroperoxidation (Table 1, entry 6), the role of CAN could be accounted for by a similar function of Mn(III) during the reaction [62,63,64].

### 2.5. Conversion of the Hydroperoxides ***2*** and ***6*** into the Alcohols ***9*** and ***10***

**Scheme 6:**
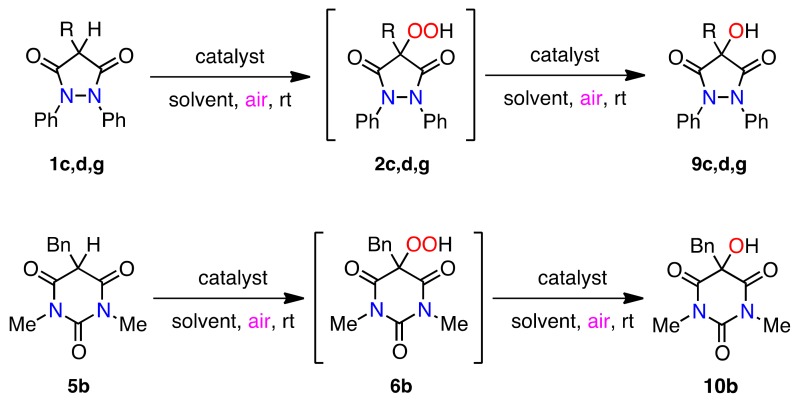
Conversion of the hydroperoxides into the corresponding alcohols.

**Scheme 7:**
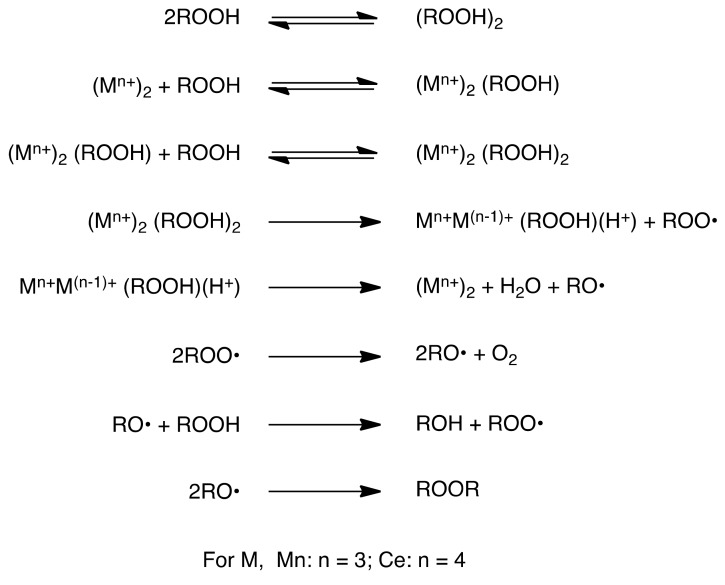
Degradation of hydroperoxides by a redox system [69].

When the aerobic oxidation of the pyrazolidinedione **1d** was carried out for a longer reaction period with a catalytic amount of Mn(OAc)_3_ in AcOH or for shorter reaction time in EtOH, a substantial amount of hydroxylated product **9d** was produced along with the hydroperoxide **2d** (Scheme 6 and Table 4, entries 2–4). Furthermore, utilizing a stoichiometric amount of CAN as a stronger oxidant than Mn(OAc)_3_ also gave a similar result (entry 5). The formation of the alcohol **9d** might be attributed to the degradation of the corresponding hydroperoxide **2d**. As we have mentioned earlier, metal ion-mediated conversion of the 1,3-dicarbonyl compounds into their corresponding 2-hydroxylated derivatives has been well studied [21,22,23,24,25,26,27,28,29,30], nevertheless the mechanism associated with this conversion was not well explored and remained more or less ambiguous. In order to scrutinize the production of **9d**, the hydroperoxide **2d** was stirred at 23 °C in AcOH without the presence of any metal catalyst and under sunlight for 23 h. As a result, no conversion of **2d** to **9d** took place and 100% recovery of **2d** was possible after the removal of the solvent (entry 6). Thus, while many alkylhydroperoxides are known to be sensitive to heat [65,66] and light [67,68], the conversion of **2d** into **9d** is not thermal or photochemical. However, in the presence of a catalytic amount of Mn(OAc)_3_ (0.01 equiv.) in the same reaction system, 47% of the **2d** was converted into its corresponding alcohol **9d** (entry 7). Carrying out a similar reaction under darkness did not alter the course of the reaction, which implies that the Mn(III)-catalyzed degradation is not light-assisted. We then carried out the reaction of **2d** under an argon atmosphere with Mn(OAc)_2_, and we found that Mn(OAc)_2_ was also effective in the degradation (entry 8). With cerium(IV), 47% of the alcohol **9d** was formed from **2d** (entry 9). A similar degradation was also observed in the reaction of the hydroperoxides **2c**, **2g** (entries 10 and 11), the barbituric acid **5b** and the hydroperoxide **6b** (entries 12–17). Therefore, we could draw the conclusion that the transformation of hydroperoxides **2** and **6** into their corresponding alcohols **9** and **10** was due to a similar redox reactions using copper salt which has been extensively studied [69,70,71]. If the Mn(III) and Ce(IV) would function such as Cu(II), the mechanism of the degradation might be depicted in Scheme 7.

**Table 4:** Aerobic oxidation of amides under various conditions followed by decomposition ^a^.

Entry	Amide	Catalyst	1:Catalyst ^b^	Solvent	Time/h	Product/yield (%) ^c^		Recovery/%
1	**1d**	Mn(OAc)_3_	1:0.1	AcOH	2	**2d** (97)		
2	**1d**	Mn(OAc)_3_	1:0.1	AcOH	12	**2d** (87)	**9d** (8)	
3	**1d**	Mn(OAc)_3_	1:0.2	AcOH	27	**2d** (52)	**9d** (46)	
4	**1d**	Mn(OAc)_3_	1:0.28	EtOH	3	**2d** (54)	**9d** (41)	
5	**1d**	CAN^d^	1:1	MeOH	1	**2d** (35)	**9d** (55)	
6	**2d**	none		AcOH	23			100
7	**2d**	Mn(OAc)_3_	1:0.01	AcOH	23		**9d** (47)	32
8^e^	**2d**	Mn(OAc)_2_	1:1	AcOH	14		**9d** (25)	66
9	**2d**	CAN^d^	1:1	MeOH	2		**9d** (47)	33
10	**2c**	Mn(OAc)_3_	1:0.01	AcOH	17		**9c** (45)	48
11	**2g**	Mn(OAc)_3_	1:0.01	AcOH	17		**9g** (40)	28
12	**5b**	Mn(OAc)_3_	1:0.1	AcOH	3	**6b** (88)	**10b** (7)	
13	**5b**	Mn(OAc)_3_	1:0.1	AcOH	5	**6b** (79)	**10b** (18)	
14	**5b**	CAN ^d^	1:1	MeOH	0.5	**6b** (47)	**10b** (43)	
15	**6b**	none		AcOH	12			100
16	**6b**	Mn(OAc)_3_	1:0.1	AcOH	14		**10b** (14)	88
17	**6b**	Mn(OAc)_3_	1:0.4	AcOH	23		**10b** (40)	50

^a^ The reaction of an amide (1 mmol) was carried out in AcOH (30 mL) at room temperature in air; ^b^ Molar ratio; ^c^ Isolated yield is based on the amide used; ^d^ The reaction with ammonium cerium(IV) nitrate (CAN) was carried out at 0 °C; ^e^ The reaction was carried out under an argon atmosphere.

### 2.6. Application of the Mn(III)-Mediated Oxygenation

**Scheme 8:**
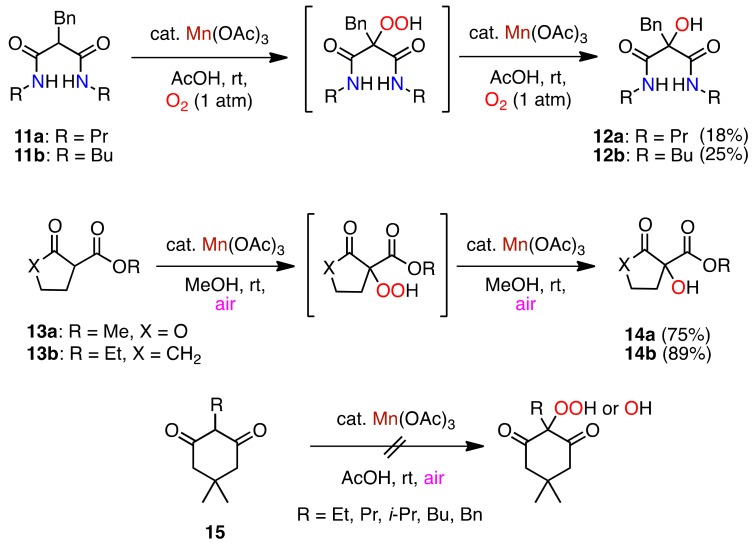
Aerobic oxidation of malonamides, cyclic *β*-diketones and *β*-ketoesters.

In order to apply the hydroperoxidation to acyclic *β*-oxoamides, cyclic ketones, and esters, the reactions of malonamides **11a**,**b**, cyclopentanonecarboxylate derivatives **13a**,**b**, and substituted dimedones **15** were examined (Scheme 8).

Surprisingly, the malonamides **11a**,**b** were inactive under the aerobic conditions and only afforded the corresponding alcohols **12a** and **12b**, respectively, under an oxygen atmosphere (1 atm) instead of air. Because the reaction of **13a**,**b** in AcOH gave an intractable mixture, the reaction was carried out in MeOH and the corresponding alcohols **14a**,**b** were isolated (Scheme 8). The dimedone derivatives **15** did not produce any oxygenated products, but **15** were recovered. Therefore, the hydroperoxidation must be characteristic for the cyclic amide derivatives. We also assumed that the corresponding hydroperoxides of **11** and **13** were too unstable in the solution containing Mn(III), and thus readily decomposed to give the corresponding alcohols **12** and **14**, respectively, according to the redox system mentioned above (Scheme 7).

The hydroperoxides **2a–d** and **6b** could be transformed by reduction into their corresponding alcohols **9a–d** and **10b**, respectively. Simple stirring of equimolar amounts of the hydroperoxide and Ph_3_P in Et_2_O at room temperature gave the corresponding alcohol after a very simple work-up procedure (see Experimental section). The results are summarized in Table 5.

**Table 5:** Reduction of the hydroperoxides **2a–d** and **6b** using Ph_3_P ^a^.

Entry	ROOH	ROOH:Ph_3_P	Time/h	Product/Yield (%) ^b^
1	**2a**	1:1	3	**9a** (96)
2	**2b**	1:1	3	**9b** (97)
3	**2c**	1:1	3	**9c** (98)
4	**2d**	1:1	3	**9d** (97)
5	**6b**	1:1	3	**10b** (80)

^a^ The reaction of the hydroperoxides **2a–d** and **6b** with Ph_3_P was carried out in Et_2_O in air at room temperature; ^b^ Isolated yield is based on **2** or **6b** used.

### 2.7. Structural Determination of the Hydroperoxides and the Hydroxyl Derivatives

**Figure 1:**
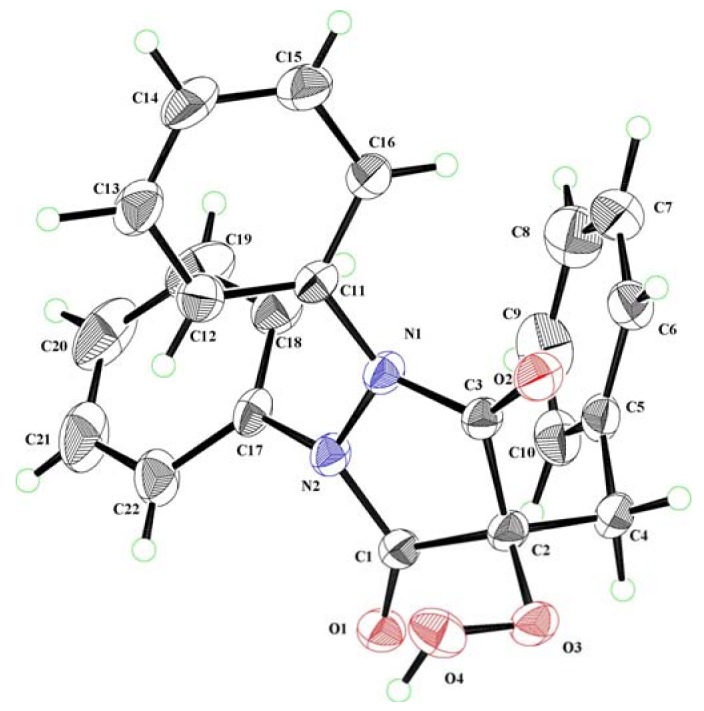
ORTEP drawing of 4-benzyl-4-hydroperoxy-1,2-diphenylpyrazolidine-3,5-dione (**2g**).

Characterization of the hydroperoxides and the alcohols deserves comment. All the obtained hydroperoxides in CH_2_Cl_2_ showed a positive potassium iodide-starch test. The structural assignment of the hydroperoxides **2**, **4**, **6**, and **8** was based on their ^1^H-NMR, ^13^C-NMR, IR spectra as well as their elemental analyses. For example, the 4-hydroperoxypyrazolidinedione **2g** showed a singlet at *δ* 11.21 ppm in the ^1^H-NMR spectrum due to OOH group. In the ^13^C-NMR spectrum, the amide carbonyl carbon appeared at *δ* 167.7 ppm and a quaternary carbon C-4 bearing the OOH group at *δ* 86.7 ppm. In addition, the elemental analysis and FAB HRMS supported the molecular formula of C_22_H_18_N_2_O_4_. The structure was finally confirmed by X-ray crystallography. A colorless single crystal of **2g** was successfully grown from CH_2_Cl_2_-benzene of approximate dimensions of 0.25 × 0.50 × 0.10 mm was mounted on a glass fiber. All measurements were made on an imaging plate diffractometer with graphite monochromated Mo-K*α* radiation. Cell constants and an orientation matrix for data collection corresponded to a primitive triclinic cell with dimensions were obtained as the triclinic space group *P-*1 with cell constants *a* = 10.3233, *b* = 10.4273, *c* = 12.9985 Å, *V* = 1283.0 Å^3^, and *α* = 95.792, *β* = 104.504, *γ* = 105.643°. The structure was solved by direct methods and expanded using Fourier techniques (see Supplementary data). The ORTEP drawing of **2g** is shown in Figure 1. The intramolecular hydrogen-bonding in **2g** could be visualized between the terminal hydroperoxy oxygen and the carbonyl oxygen, O(4)-O(1) (2.705 Å) [32,33,37,38,39,40,42]. The other hydroperoxides obtained from the aerobic oxidation showed similar spectroscopic features.

In the case of the alcohol derivatives **9**, **10**, **12**, and **14**, the characteristic spectral features of the OH group were observed in the IR and NMR spectra. For example, **9g** showed an absorption band at 3298 cm^−1^ in the IR spectrum corresponding to the OH group and a singlet at *δ* 4.88 ppm in the ^1^H-NMR spectrum due to the OH group. In the ^13^C-NMR spectrum, the quaternary carbon C-4 bearing the OH group appeared at *δ* 75.8 ppm and this is significantly different from that having the OOH group (Table 6) [72]. In addition, the elemental analysis of **9g** supported the molecular formula of C_22_H_18_N_2_O_3_. Therefore, it is easy to distinguish the corresponding alcohols from the hydroperoxides.

**Table 6:** ^13^C-NMR chemical shifts of the quaternary carbon bearing the OOH and OH group ^a^.

Hydroperoxide		^13^C-OOH/ppm	Alcohol		^13^C-OH/ppm
**2a**	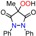	81.6	**9a**	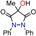	71.1
**2b**	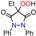	85.8	**9b**	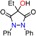	74.7
**2c**	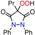	85.3	**9c**	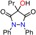	74.0
**2d**	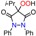	87.6	**9d**	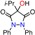	76.3
**2g**	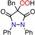	86.7	**9g**	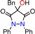	75.8
**6b**	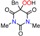	87.2	**10b**	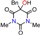	76.6

^a^ The ^13^C-NMR spectrum was measured in CDCl_3_.

## 3. Experimental

### 3.1. Measurements

Melting points were taken using a Yanagimoto micromelting point apparatus and were not corrected. The NMR spectra were recorded using a JNM AL300 or ECX 500 FT-NMR spectrometer at 300 or 500 MHz for ^1^H and 75 or 125 MHz for ^13^C, with tetramethylsilane as the internal standard. The chemical shifts are reported in *δ* values (ppm) and the coupling constants in Hz. The IR spectra were measured in CHCl_3_ or KBr using a Shimadzu 8400 FT IR spectrometer and expressed in cm^−1^. The EI MS spectra were measured by a Shimadzu QP-5050A gas chromatograph-mass spectrometer with the ionizing voltage of 70 eV. The high-resolution mass spectra and the elemental analysis were performed at the Instrumental Analysis Center, Kumamoto University, Kumamoto, Japan.

### 3.2. Materials

Manganese(II) acetate tetrahydrate, Mn(OAc)_2_•4H_2_O, was purchased from Wako Pure Chemical Ind., Ltd. Manganese(III) acetate dihydrate, Mn(OAc)_3_•2H_2_O, was prepared according to the method described in the literature [73,74]. 4-Alkyl-1,2-diphenylpyrazolidine-3,5-diones **1a–h** were prepared by the condensation of a suitable alkylmalonate with 1,2-diphenylhydrazine in the presence of NaH in boiling chlorobenzene [17]. Methyl 2-oxotetrahydrofuran-3-carboxylate (**13a**) and ethyl 2-oxo- cyclopentanecarboxylate (**13b**) were purchased from Tokyo Chemical Industry Co., Ltd., and used as received. The physical data for the pyrazolidinediones **1a–h** are given in Supplementary data.

1,3-Dialkyl-substituted pyrrolidine-2,4-diones **3a-s** were prepared as follows [75,76,77,78,79,80,81]. To a mixture of ethyl (benzylamino)acetate (3.22 mL; 17.4 mmol) and triethylamine (4.85 mL; 34.8 mmol) in CHCl_3_ (25 mL) was dropwise-added propanoyl chloride (1.65 mL; 19.2 mmol) at 0 °C over 15 min. After stirring for another 1.5 h at room temperature, the mixture was diluted with CHCl_3_ to 50 mL and washed with a 5% aqueous AcOH solution (25 mL), water (50 mL), brine (25 mL), dried over anhydrous MgSO_4_, and then concentrated to dryness, affording the liquid propanoyl-protected (benzylamino)acetate with sufficient purity for use in the next step.

To a refluxing suspension of NaH (60% dispersion in mineral oil) (500 mg; 11.03 mmol) and tetrahydrofuran (50 mL) in a 300 mL three-necked flask was dropwise-added the prepared propanoyl-protected (benzylamino)acetate (2.5 g; 10.03 mmol) in tetrahydrofuran (50 mL). After this addition, the mixture was continuously heated under reflux for 12 h. A pale yellow solid was formed during the heating and then filtered under suction. The obtained solid was dissolved in a minimum volume of water and very carefully acidified with 2 M H_2_SO_4_, giving 1-benzyl-3-methyl-2,4-pyrrolidinedione (keto form of **3a**) as a crude precipitate. The precipitate was purified by silica gel column chromatography, eluting with a mixture of EtOAc and hexane followed by recrystallization using the appropriate solvent. The other 2,4-pyrrolidinediones **3b–s** were prepared in a manner similar to that described above. The physical data for the pyrrolidinediones **3a–s** are given in the Supplementary Data.

5-Alkyl-1,3-dimethylbarbituric acids **5a–e** were prepared as follows [49]: 1,3-Dimethylbarbituric acid (5 mmol) and 10%Pd-C (0.2 g) were added to a mixture of acetone (20 mL), AcOH (20 mL), and water (10 mL). A few drops of concentrated H_2_SO_4_ were added and the mixture was heated at 50 °C under hydrogen (2 atm) for 4 h using a glass reactor. The mixture was filtered in a Celite column and the filtrate was extracted with CHCl_3_. The extract was washed with water several times and a saturated aqueous solution of NaHCO_3_, and then dried over anhydrous MgSO_4_. After removal of the solvent, the crude product was recrystallized from Et_2_O/hexane, giving 1,3-dimethyl-5-*i*-proprylbarbituric acid (**5a**) in 90% yield. A similar reaction of the barbituric acid (5 mmol) with 2-naphthaldehyde (5 mmol) was carried out in MeOH (30 mL) at the reflux temperature for 10 h. After the same work-up, 5-(2-naphthyl)methyl-1,3-dimethylbarbituric acid **5e** was obtained in 88% yield. Other benzyl-substituted barbituric acids **5b–d** were prepared in the same manner. The physical data for the barbituric acids **5a–e** are given in Supplementary data.

3-Butyl-4-hydroxy-2-quinolinone **7** was prepared according to the standard procedure described in the literature [50,51,52]. Aniline (2.80 g; 30 mmol) and diethylbutyl malonate (6.51 g; 30 mmol) were heated at 150 °C for 20 h. Water (30 mL) was added and the mixture was extracted with CH_2_Cl_2_. The extract was washed with 2 M HCl, water and dried over anhydrous Na_2_SO_4_. After removal of the solvent, ethyl 2-(phenylcarbamoyl)hexanoate (28 mmol) was obtained. The hexanoate in EtOH (10 mL) was hydrolyzed with 6 M NaOH (20 mL) in EtOH (20 mL) at room temperature for 1 h. After acidifying the solution with 2 M HCl, the precipitates were filtered with suction. The obtained solid acid was dehydrated in PPA, which was poured into water and then neutralized using 6 M NaOH to pH 4, giving **7** as a precipitate (3.67 g; 56%). The physical data for the quinolinone **7** are given in the Supplementary Data.

2-Benzyl-*N*^1^,*N*^3^-dipropylmalonamide (**11a**) and 2-benzyl-*N*^1^,*N*^3^-dibutylmalonamide (**11b**) were prepared by the reaction of diethyl 2-benzylmalonamide (2.50 g) with propylamine and butylamine (50 mL), respectively, at reflux temperature for 24 h followed by removal of the amine unchanged under reduced pressure to give a colorless solid which was recrystallized with CHCl_3_/hexane [82]. The physical data for the malonamides **11a** and **11b** are given in the Supplementary Data.

### 3.3. Mn(III)-Initiated Aerobic Oxidation of Heterocyclic 1,3-Dicarbonyl Compounds ***1a–h**, **3a–p**, **5a–e**,* and ***7***

A typical procedure is as follows: to a solution of the cyclic amide (1 mmol) in glacial AcOH (25 mL), Mn(OAc)_3_•2H_2_O (26.8 mg; 0.1 mmol) was added. The mixture was stirred at room temperature in air for 1.5–2 h, and then the reaction was quenched by adding water (25 mL) to the mixture. The aqueous reaction mixture was extracted three times with CH_2_Cl_2_ (30 mL) and the combined extract was washed with water, then a saturated aqueous solution of NaHCO_3_, dried over anhydrous MgSO_4_, and concentrated to dryness. Although the product was almost pure, it was further purified by silica gel flash column chromatography, eluting with Et_2_O/hexane (7:3 v/v) or EtOAc/hexane (8:2 v/v) if needed. The physical data for the hydroperoxides **2a–h**, **4a–p**, **6a–e**, and **8** are given in the Supplementary Data.

### 3.4. Conversion of the Hydroperoxides ***2*** and ***6*** into the Alcohols ***9*** and ***10***

The redox reaction of the hydroperoxide **2c**, **2d**, **2g**, **6b** (1 mmol) was carried out in AcOH (30 mL) at room temperature in air. The molar ratio of the catalyst and the reaction times are described in Table 4. After the usual work-up, the corresponding alcohols **9c**, **9d**, **9g**, and **10b** were obtained. On the other hand, the reduction of the hydroperoxides **2a–d** and **6b** using Ph_3_P is as follows: to the hydroperoxide (0.5 mmol) in Et_2_O (10 mL), a solution of Ph_3_P (0.5 mmol) in Et_2_O (10 mL) was dropwise added and stirred for 3 h at room temperature. Water (20 mL) was added to the reaction mixture and then extracted with CH_2_Cl_2_. The extract was dried over anhydrous Na_2_SO_4_ and then evaporated to dryness. Simple recrystallization from Et_2_O/hexane gave the pure crystalline alcohol. The physical data for the alcohols **9a–d**, **9g**, and **10b** are given in the Supplementary Data.

### 3.5. Aerobic Oxidation of ***1d***, ***2d***, and ***5b*** Using Ammonium Cerium (IV) Nitrate (CAN)

A typical procedure is as follows: to an ice-cooled solution of the substrate **1d**, **2d**, or **5b** (1 mmol) in MeOH (20 mL), a solution of ammonium cerium(IV) nitrate (CAN) (1 mmol) in MeOH (10 mL) was dropwise-added and stirred in air until the orange color of Ce(IV) disappeared. The reaction was quenched by adding water (25 mL). The aqueous reaction mixture was extracted three times with CH_2_Cl_2_ (30 mL), and the combined extract was washed with water, saturated brine, dried over anhydrous Na_2_SO_4_, and then concentrated to dryness. The residue was separated by a silica gel column, eluted with EtOAc/hexane (7:3 v/v). The obtained products **2d**, **9d**, **6b**, and **10b** were further purified by recrystallization from the solvent described above.

### 3.6. Mn(III)-Initiated Aerobic Oxidation of Acyclic Amides ***11a***,***b***, ketones ***13b***, and Esters ***13b***,***15***

The oxidation of **11a** and **11b** (0.7 mmol) was carried out at room temperature in glacial AcOH (10 mL) in the presence of Mn(OAc)_3_•2H_2_O (0.42 mmol) under an oxygen atmosphere (1 atm) for 29 h. After the usual work-up, the corresponding alcohols **12a** and **12b** were obtained in poor yields. The physical data for the alcohols **12a** and **12b** are given in the Supplementary Data.

The aerobic oxidation of cyclopentanonecarboxylate derivatives **13a** and **13b** was as follows. To a solution of the cyclopentanonecarboxylate (1 mmol) in MeOH (10 mL), a mixture of Mn(OAc)_3_•2H_2_O (0.1 mmol) in MeOH (20 mL) was dropwise-added, and the mixture was stirred at room temperature for 12 h in air. The reaction mixture was then filtered through the silica gel column (eluted with Et_2_O) to remove the catalyst. After removal of all volatile components using an evaporator, the corresponding alcohols **14a** and **14b** were obtained as oil. The physical data for the alcohols **14a** and **14b** are given in the Supplementary Data.

2-Alkyl-5,5-dimethylcyclohexane-1,3-diones **15** (R = Et, Pr, *i*-Pr, Bu, Bn) were prepared by the reaction of dimedone with the corresponding alkylbromide in the presence of sodium ethoxide in boiling ethanol [83]. The Mn(III)-catalyzed aerobic oxidation of **15** was carried out under various conditions; however, no reaction occurred.

## 4. Conclusions

The facile *α*-oxygenation of cyclic amides was achieved by Mn(III)-initiated aerobic oxidation under very mild conditions. The direct hydroperoxidation was characteristic for the cyclic amides, such as 4-alkyl-1,2-diphenylpyrazolidine-3,5-diones **1a–h**, 3-alkylpyrrolidine-2,4-diones **3a–p**, 3-alkyl-1,5-dimethylbarbituric acids **5a–e**, and 3-butyl-4-hydroxy-2-quinolinone **7**. It was found that the obtained hydroperoxides gradually underwent redox degradation in the presence of Mn(III)/Mn(II) or Ce(IV)/Ce(III) under the conditions to convert into the corresponding alcohols. Acyclic amides and esters, such as malonamides **11a**,**b** and cyclopentanonecarboxylates **13a**,**b**, afforded the alcohols **12a**,**b** and **14a**,**b**, probably derived from a similar redox degradation of the corresponding hydroperoxides. Cyclic diketones, such as dimedone **15**, was inactive under the aerobic oxidation conditions. The reason why the hydroperoxides were isolated in this reaction is assumed to be that, (1) it is difficult for the nucleophilic attack of the hydroperoxy group to occur at the amide carbonyl because the electrophilicity of the amide carbonyl carbon should be weak, and (2) the hydroperoxy group is stabilized by intramolecular hydrogen-bonding with the carbonyl group. The hydroperoxides are easily transformed into the corresponding alcohols by normal reduction using Ph_3_P.

Antimalarial testing of the hydroperoxide **2g** was also performed using *Plasmodium falciparum* FCR-3 strain (ATCC 30932), and a weak antimalarial activity (14% inhibition) was confirmed. In addition, in order to examine an inhibitory effect toward mRNAs in cells, HeLa cells were treated with the hydroperoxide **2g** or **6b**. However, an appreciable effect on the intracellular distribution of mRNAs was not observed (data not shown).

## Supplementary Materials

Supplementary materials can be accessed on: http://www.mdpi.com/1420-3049/16/11/9562/s1.

## References

[1] Chen B.-C., Zhou P., Davis F.A., Ciganek E. (2003). *a*-Hydroxylation of enolates and silyl enol ethers. Org. React..

[2] Vedej E., Engler D.A., Telschow J.E.  (1978). Transition-metal peroxide reactions. Synthesis of *α*-hydroxycarbonyl compounds from enolates. J. Org. Chem..

[3] Abu-Omar M.M., Espenson J.H. (1996). Oxidations of cyclic *β*-diketones catalyzed by methylrhenium trioxide. Organometallics.

[4] Heathcock C.H., Mahain C., Schlecht M.F., Utawanit T. (1984). A synthetic approach to the quassinoids. J. Org. Chem..

[5] Davis F.A., Clark C., Kumar A., Chen B.-C. (1994). Asymmetric synthesis of the AB ring segments of daunomycin and 4-demethoxydaunomycin. J. Org. Chem..

[6] Davis F.A., Liu H., Chen B.-C., Zhou P. (1998). Oxidation of 1,3-dicarbonyl compounds using (camphorylsulfonyl)oxaziridines. Tetrahedron.

[7] Zhang W., Watanabe K., Cai X., Jung M.E., Tang Y., Zhan J. (2008). Identifying the minimal enzymes required for anhydrotetracycline biosynthesis. J. Am. Chem. Soc..

[8] Giovanelli E., Leroux S., Moisan L., Carreyre H., Thuéry P., Buisson D.-A., Meddour A., Coustard J.-M., Thibaudeau S., Rousseau B. (2011). On the elucidation of the mechanism of *Vinca* alkaloid fluorination in superacidic medium. Org. Lett..

[9] Büchi G., Matsumoto K.E, Nishimura H.  (1971). Total synthesis of (±)-vindorosine. J. Am. Chem. Soc..

[10] Andriamialisoa R.Z., Langlois N., Langlois Y. (1985). A new efficient total synthesis of vindorosine and vindoline. J. Org. Chem..

[11] Sasaki Y., Kato D., Boger D.L. (2010). Asymmetric total synthesis of vindorosine, vindoline, and key vinblastine analogues. J. Am. Chem. Soc..

[12] Rannoux C., Roussi F., Martin M.-T., Guéritte F. (2011). Elaboration of vinblastine hybrids using a reactive in situ generated *N*-carboxyanhydride. Org. Biomol. Chem..

[13] Zhu J., Klunder J.H., Zwanenburg B. (1994). Stereospecific total synthesis of (−)-kjellmanianone and a revision of its absolute configuration. Tetrahedron Lett..

[14] Charest M.G., Lerner C.D., Brubaker J.D., Siegel D.R., Myers A.G. (2005). A convergent enantioselective route to structurally diverse 6-deoxytetracycline antibiotics. Science.

[15] Mark G., Charest M.G., Siegel D.R., Myers A.G. (2005). Synthesis of (−)-tetracycline. J. Am. Chem. Soc..

[16] Reed G.A., Griffin I.O., Eling T.E. (1985). Inactivation of prostaglandin H synthase and prostacyclin synthase by phenylbutazone. Requirement for peroxidative metabolism. Mol. Pharmacol..

[17] Vennerstorm J.L., Holmes T.J. (1987). Preparation and evaluation of electrophilic derivatives of phenylbutazone as inhibitors of prostaglandin-*H*-synthase. J. Med. Chem..

[18] Mentz V.P., Schulz M., Kluge R.  (1987). Chemische darstellung von phenylbutazon-hydroperoxid und prüfung der substanz auf herzwirksamkeit unter *in-vitro-* und *in-vivo-*bedingungen. Arzneim.-Frosch..

[19] Rechenberg H.K.V. (1962). Phenylbutazone.

[20] Chauhan S.M.S., Srinivas K.A., Mohapatra P.P.  (1999). Oxidation of phenylbutazone with hydrogen peroxide catalyzed by 5,10,15,20-tetraarylporphyrinoiron(III) chlorides in dichloromethane. Ind. J. Chem.

[21] Demir A.S., Jeganathan A. (1992). Selective oxidation of *α*,*β*-unsaturated ketones at the *α*′-position. Synthesis.

[22] Vedejs E., Engler D.A., Telschow J.E. (1978). Transition-metal peroxide reactions. Synthesis of *α*-hydroxycarbonyl compounds from enolates. J. Org. Chem.

[23] Hubert A.J., Starcher P.S. (1968). The Baeyer-Villiger oxidation of alkyl oxocyclohexanecarboxylates. J. Chem. Soc. C.

[24] Adam W., Smerz A.K. (1996). Nickel-catalyzed hydroxylation of 1,3-dicarbonyl compounds by dimethyldioxirane. Tetrahedron.

[25] Christoffers J. (1999). Novel manganese-catalyzed α-oxidation of cyclic *β*-keto esters with molecular oxygen. J. Org. Chem..

[26] Christoffers J., Wenner T. (2002). Cerium-catalyzed *α*-oxidation of *β*-dicarbonyl compounds with molecular oxygen. SynLett.

[27] Christoffers J., Werner T., Unger S., Frey W. (2003). Preparation of acyloins by cerium-catalyzed, direct hydroxylation of *β*-dicarbonyl compounds with molecular oxygen. Eur. J. Org. Chem..

[28] Baucherel X., Levoirier E., Uziel J., Juge S. (2000). Monohydroxylation of cyclic and acyclic *β*-keto esters with molecular oxygen catalyzed by cobalt(II) chloride in neutral conditions. Tetrahedron Lett..

[29] Nair V., Nair L.G., Mathew J. (1998). Cerium(IV) mediated oxygenation of dialkyl malonates: A novel synthesis of tartronic acid derivatives. Tetrahedron Lett..

[30] Watanabe T., Ishikawa T. (1999). Mild air-oxidation of 1,3-dicarbonyl compounds with cesium salts: Novel *α*-hydroxylation accompanied by partial hydrolysis of malonate derivatives. Tetrahedron Lett..

[31] Nishino H. (1985). The facile synthesis of dihydrofurans by the oxidation of olefins with tris(2,4-pentanedionato)manganese(III). Bull. Chem. Soc. Jpn..

[32] Tategami S., Yamada T., Nishino H., Korp J.D., Kurosawa K. (1990). Formation of 1,2-dioxacyclohexanes by the reaction of alkenes with tris(2,4-pentanedionato)manganese(III) or with manganese(III) acetate. Tetrahedron Lett..

[33] Nishino H., Tategami S., Yamada T., Korp J.D., Kurosawa K. (1991). Formation of 1, 2-dioxanes by the use of tris (2,4-pentanedionato)-manganese (III) or manganese (III) acetate. Bull. Chem. Soc. Jpn..

[34] Asahi K., Nishino H. (2008). Facile endoperoxypropellane synthesis by manganese(III) acetate-mediated aerobic oxidation. Eur. J. Org. Chem..

[35] Tsubusaki T., Nishino H. (2009). Formation of 1,2-dioxolanes using Mn(III)-based reaction of various arylacetylenes with 2,4-pentanedione and related reaction. Tetrahedron.

[36] Haque M.A., Nishino H. (2011). Synthesis of peroxylactones using Mn(III)-catalyzed aerobic oxidation. Heterocycles.

[37] Qian C.-Y., Nishino H., Kurosawa K. (1993). Manganese(II) acetate-mediated double 2-hydroperoxyalkylations of barbituric acid and its derivatives. J. Org. Chem..

[38] Rahman M.T., Nishino H., Qian C.-Y. (2003). Synthesis of 4,4-bis(2-hydroperoxyalkyl)pyrazolidine-3,5-diones using manganese(III)-catalyzed autoxidation. Tetrahedron Lett..

[39] Rahman M.T., Nishino H. (2003). Manganese(III)-based oxidation of 1,2-disubstituted pyrzolidine-3,5-diones in the presence of alkenes. Tetrahedron.

[40] Kumabe R., Nishino H. (2004). A unique peroxide formation based on the Mn(III)-catalyzed aerobic oxidation. Tetrahedron Lett..

[41] Haque M.A., Nishino H. (2011). Synthesis of peroxylactones using Mn(III)-catalyzed aerobic oxidation. Heterocycles.

[42] Rahman M.T., Nishino H. (2003). Manganese(III)-catalyzed facile direct hydroperoxidation of some heterocyclic 1,3-dicarbonyl compounds. Org. Lett..

[43] Fugger J., Tien J.M., Hunsberger I.M. (1955). The preparation of substituted hydrazines. I. Alkylhydrazines via alkylsydnone. J. Am. Chem. Soc..

[44] King J.A., McMillan F.H. (1950). The preparation of some *α*-benzylamino-*β*,*β*-dialkoxypropionic acid derivatives. J. Am. Chem. Soc..

[45] Koech P.K., Krische M.J. (2004). Catalytic addition of metallo-aldehyde enolates to ketones: A new C−C bond-forming hydrogenation. Org. Lett..

[46] Speziale A.J., Jaworski E.G. (1960). *N*-Substituted glycinate and alaninate esters. J. Org. Chem..

[47] Zhu Y., Zou X., Hu F., Yao C., Liu B., Yang H. (2005). Synthesis and herbicidal evaluation of novel 3-[(*α*-Hydroxy-substituted)benzylidene]pyrrolidine-2,4-diones. J. Agric. Food Chem..

[48] Chowdhury F.A., Nishino H., Kurosawa K. (1999). Simple route to azabicyclic peroxides from tetramic acid derivatives usingmanganese(III)-based molecular oxygen trapping reaction. Heterocycles.

[49] Jursic B.S., Neumann D.M. (2001). Reductive *C*-alkylation of barbituric acid derivatives with carbonyl compounds in the presence of platinum and palladium catalysts. Tetrahedron Lett..

[50] Detsi A., Bardakos V., Markopoulos J., Igglessi-Markopoulou O. (1996). Reactions of 2-methyl-3,1-benzoxazin-4-one with active methylene compounds: A new route to 3-substituted 4-hydroxyquinolin-2(1*H*)-ones. J. Chem. Soc. Perkin Trans. 1.

[51] McQuaid L.A., Smith E.C., Lodge D., Pralong E., Wikel J.H., Calligaro D.O., O'Malley P.J. (1992). 3-Phenyl-4-hydroxyquinolin-2(1*H*)-ones: Potent and selective antagonists at the strychnine-insensitive glycine site on the *N*-methyl-D-aspartate receptor complex. J. Med. Chem..

[52] Klásek A., Mrkvicka V., Pevec A., Kosmrlj J.  (2004). Novel tandem hydration/cyclodehydration of *α*-thiocyanatoketones to 2-oxo-3-thiazolines. Application to thiazolo[5,4-*c*]quinoline-2,4(3a*H*,5*H*)-dione Synthesis. J. Org. Chem.

[53] Ohshima T., Sodeoka M., Shibasaki M. (1993). Manganese(III)-based oxidative free-radical reaction of *α*-allyl-*β*-keto ester with molecular oxygen. Tetrahedron Lett..

[54] Lamarque L., Méou A., Brun P. (2000). Mn(OAc)_3_-promoted hydroxylation of *α*-carbomethoxy-*γ*-lactones by molecular oxygen. Can. J. Chem..

[55] Lide D.R. (1994). Handbook of Chemistry and Physics.

[56] Ling K.-Q., Lee Y., Macikenas D., Protasiewicz J.D., Sayre L.M. (2003). Copper(II)-mediated autoxidation of *tert*-butylresorcinols. J. Org. Chem.

[57] Bredereck H., Bauer G. (1970). Hydroperoxide cyclischer *β*-diketone. JustusLiebigs Ann. Chem..

[58] Tona M., Guardiola M., Fajari L., Messeguer A. (1995). A study on the mechanism and scope of the radical-mediated oxidation of arylacetoacetates. Tetrahedron.

[59] Nair V., Nair L.G., Mathew S. (1998). Cerium(IV) mediated oxygenation of dialkyl malonates: A novel synthesis of tartronic acid derivatives. Tetrahedron Lett..

[60] Neumann B., Müller S.C., Hauser M.J.B., Steinbock O., Simoyi R.H., Dalal N.S. (1995). Identification and kinetics study of the peroxymalonyl radical in the aerobic oxidation of malonic acid by cerium(IV). J. Am. Chem. Soc..

[61] Yoshioka M., Nishioka T., Hasegawa T. (1993). Dye-sensitized photooxidation of 6-acyl- and 6-carboalkoxybenzocycloalken-5-ones: Reaction of singlet oxygen with enolic 1,3-dicarbonyl compounds. J. Org. Chem..

[62] Christoffers J., Wenner T. (2002). Cerium-catalyzed *α*-oxidation of *β*-dicarbonyl compounds with molecular oxygen. SynLett.

[63] Christoffers J., Werner T., Unger S., Frey W. (2003). Preparation of acyloins by cerium-catalyzed, direct hydroxylation of *β*-dicarbonyl compounds with molecular oxygen. Eur. J. Org. Chem..

[64] Ye J.-H., Xue J., Ling K.-Q., Xu J.-H. (1999). Ceric ammonium nitrate (CAN) mediated novel dimerizations of 4-hydroxyquinolin-2(1*H*)-ones: The first example of reactions of oxygen-centered radicals from 1,3-dicarbonyl compounds. Tetrahedron Lett..

[65] Hiatt R., Irwin K.C. (1968). Homolytic decompositions of hydroperoxides. V. Thermal decompositions. J. Org. Chem..

[66] Stannett V., Mesrobian R.B. (1950). The kinetics of the decomposition of tertiary hydroperoxides in solvents. J. Am. Chem. Soc..

[67] Boyaci F.G., Takaç S., Özdamar T.H.  (2000). Effects of process parameters on the kinetics of the decomposition of 2-isopropylnaphthalene hydroperoxide into 2-naphthol and acetone. Rev. Chem. Engl..

[68] Dannley R.L., Jalics G. (1965). The decomposition of silyl hydroperoxides. J. Org. Chem..

[69] Richardson W.H.  (1966). Metal ion decomposition of hydroperoxides. IV. Kinetics and products of copper salt catalyzed decomnposition of *t*-butyl hydroperxide. J. Am. Chem. Soc..

[70] Sheldon R.A., Kochi J.K. (1981). Metal Catalyzed Oxidations in Organic Chemistry.

[71] Hirano K., Sakaguchi S., Ishii Y. (2002). Radical addition of ethers to alkenes under dioxygen catalyzed by *N*-hydroxyphthalimide (NHPI)/Co(OAc)_2_. Tetrahedron Lett..

[72] Olah G.A., Parker D.G., Yoneda N., Pelizza F.  (1976). Oxyfunctionalization of hydrocarbons. 1. Protolytic cleavage-rearrangement reactions of tertiary alkyl hydroperoxides with magic acid. J. Am. Chem. Soc..

[73] Andrulis P.J., Dewar M.J.S., Dietz R., Hunt R.L.  (1966). Aromatic oxidation by electron transfer. I. Oxidations of *p*-methoxytoluene. J. Am. Chem. Soc..

[74] Heiba E.I., Dessau R.M., Koehl W.J. (1969). Oxidation by metal salts. III. Reaction of manganic acetate with aromatic hydrocarbons and the reactivity of the carboxymethyl radical. J. Am. Chem. Soc..

[75] Fugger J., Tien J.M., Hunsberger I.M. (1955). The preparation of substituted hydrazines. I. Alkylhydrazines via alkylsydnone. J. Am. Chem. Soc..

[76] King J.A., McMillan F.H. (1950). The preparation of some *α*-benzylamino-*β*,*β*-dialkoxypropionic acid derivatives. J. Am. Chem. Soc..

[77] Koech P.K., Krische M.J. (2004). Catalytic addition of metallo-aldehyde enolates to ketones: A new C−C bond-forming hydrogenation. Org. Lett..

[78] Speziale A.J., Jaworski E.G. (1960). *N*-Substituted glycinate and alaninate esters. J. Org. Chem..

[79] Zhu Y., Zou X., Hu F., Yao C., Liu B., Yang H. (2005). Synthesis and herbicidal evaluation of novel 3-[(*α*-hydroxy-substituted)benzylidene]pyrrolidine-2,4-diones. J. Agric. Food Chem..

[80] Lee V.J., Branfman A.R., Herrin T.R., Rinehart K.L. (1978). Synthesis of 3-dienoyl tetramic acids related to streptolydigin and tirandamycin. J. Am. Chem. Soc..

[81] Mallinger A., Nadal B., Chopin N., Gall T.L. (2010). One-pot synthesis of 3-aryltetramic acids. Eur. J. Org. Chem..

[82] Nemoto H., Kubota Y., Yamamoto Y. (1990). Development of a new acyl anion equivalent for the preparation of masked activated esters, and their use to prepare a dipeptide. J. Org. Chem..

[83] Martínez R., Clara-Sosa A., Apan M.T.R. (2007). Synthesis and cytotoxic evaluation of new (4,5,6,7-tetrahydro-indol-1-yl)-3-*R*-propionic acids and propionic acid ethyl esters generated by molecular mimicry. Bioorg. Med. Chem..

