# Supplementary material for: Mn(III)-Initiated Facile Oxygenation of Heterocyclic 1,3-Dicarbonyl Compounds
Source: Molecules. 2011 Nov 16;16(11):9562–81. doi: 10.3390/molecules16119562 (PMC6264282; doi:10.3390/molecules16119562)
Supplement: Supplementary file 1 [file molecules-16-09562-s001.docx]

**Supplementary Data**

The physical data for the pyrazolidinediones **1a**–**h** are given below.

*4-Methyl-1,2-phenylpyrazolidine-3,5-dione* (**1a**). Colorless needles (from CHCl_3_-hexane); mp 111–113 °C; IR (KBr) ** 1751, 1716 (C=O); ^1^H-NMR (300 MHz, CDCl_3_) *δ* 7.32–7.12 (10H, m, arom H), 3.37 (1H, q, *J* = 7.7 Hz, -CH-), 1.55 (3 H, d, *J* = 7.7 Hz, Me); ^13^C-NMR (75 MHz, CDCl_3_) *δ* 170.7 (C=O), 135.6 (arom C), 128.9, 126.7, 122.4 (arom CH), 41.1 (C-4), 12.0 (Me). Anal. Calcd for C_16_H_14_N_2_O_2_: C, 72.16; H, 5.30; N, 10.52. Found: C, 72.01; H, 5.18; N, 10.39.

*4-Ethyl-1,2-diphenylpyrazolidine-3,5-dione* (**1b**). Colorless microcrystals (from CH_2_Cl_2_-hexane); mp 104 °C (lit, mp 105–106 °C); IR (KBr) ** 1751, 1710 (C=O); ^1^H-NMR (300 MHz, CDCl_3_) *δ* 7.35–7.12 (10H, m, arom H), 3.34 (1H, t, *J* = 5.4 Hz, -CH-), 2.15–2.09 (2H, m, -CH_2_-CH_3_) 1.06 (3H, t, *J* = 7.5 Hz,
-CH_2_-CH_3_); ^13^C-NMR (75 MHz, CDCl_3_) *δ* 170.1 (C=O), 135.6 (arom C), 128.8, 126.6, 122.4 (arom CH), 47.1 (C-4), 21.6 (-CH_2_-CH_3_, Et), 10.1 (-CH_2_-CH_3_, Et).

*1,2-Dipheny-4-propylpyrazolidine-3,5-dione* (**1c**).Colorless microcrystals (from CHCl_3_-hexane); mp 103 °C; IR (KBr) ** 1753, 1720(C=O); ^1^H-NMR (300 MHz, CDCl_3_) *δ* 7.31–7.13 (10H, m, arom H), 3.39 (1H, t, *J* = 6.0 Hz, -CH-), 2.10–2.02 (2H, m, -CH_2_-C_2_H_5_), 1.58–1.50 (2H, m, -CH_2_-CH_2_-CH_3_), 0.98–0.93 (3H, m, -CH2-CH_2_-CH_3_,); ^13^C-NMR (75 MHz, CDCl_3_) *δ* 170.4 (C=O), 135.7 (arom C), 128.9, 126.7, 122.5 (arom CH), 46.1 (C-4), 30.1 (-CH_2_-CH_2_-CH_3_), 19.2 (-CH_2_-CH_2_-CH_3_), 13.8 (-CH_2_-CH_2_-CH_3_). Anal. Calcd for C_18_H_18_N_2_O_2_: C, 73.45; H, 6.16; N, 9.52. Found: C, 73.46; H, 6.06; N, 9.43.

*1,2-Diphenyl-4-*i*-propylpyrazolidine-3,5-dione* (**1d**). Colorless needles (from Et_2_O-hexane); mp 142 °C (lit, mp 144–145 °C); IR (KBr) * *1749, 1714 (C=O); *δ* 7.31–7.13 (10H, m, arom H), 3.24 (1H, d,  *J* = 3.6 Hz, -CH-), 2.65–2.58 (1H, m, CH_3_-CH-CH_3_), 1.19 (6H, d, *J* = 6.9 Hz, CH_3_-CH-CH_3_);  ^13^C-NMR (75 MHz, CDCl_3_) *δ* 169.8 (C=O), 135.7 (arom C), 128.9, 126.7, 122.5 (arom CH), 51.8 (C-4), 30.6 (CH_3_-CH-CH_3_), 19.0 (CH_3_-CH-CH_3_). Anal. Calcd for C_18_H_18_N_2_O_2_: C, 73.45; H, 6.16; N, 9.52. Found: C, 73.37; H, 6.12; N, 9.49.

*4-Butyl-1,2-diphenylpyrazolidine-3,5-dione* (**1e**).Colorless needles; mp 104–106 °C (from EtOH) (lit, mp 105.5–106.5 °C); IR (KBr) ** 1751, 1716 (C=O); ^1^H-NMR (300 MHz, CDCl_3_) *δ* 7.35–7.15 (10H, m, arom H), 3.40–3.36 (1H, m, -CH-), 2.12–2.04 (2H, m, -CH_2_-C_3_H_7_), 1.51–1.32 (4H, m, -CH_2_-CH_2_-CH_2_-CH_3_), 0.92–0.87 (3H, m, -CH_2_-CH_2_-CH_2_-CH_3_); ^13^C-NMR (75 MHz, CDCl_3_) *δ* 170.3 (C=O), 135.7 (arom C), 128.9, 126.7, 122.5 (arom CH), 46.2 (C-4), 27.9 (-CH_2_-CH_2_-CH_2_-CH_3_), 22.4 (-CH_2_-CH_2_-CH_2_-CH_3_ and -CH_2_-CH_2_-CH_2_-CH_3_), 13.6 (-CH_2_-CH_2_-CH_2_-CH_3_).

*4-t-Butyl-1,2-diphenylpyrazolidine-3,5-dione* (**1f**).Colorless needles (from Et_2_O-hexane); mp 178 °C; IR (KBr) ** 1743, 1708 (C=O); ^1^H-NMR (300 MHz, CDCl_3_) *δ* 7.32–7.12 (10H, m, arom H), 2.95 (1H, s, -CH-), 1.25 (9H, s, 3 × CH_3_); ^13^C-NMR (75 MHz, CDCl_3_) *δ* 169.4 (C=O), 135.8 (arom C), 128.8, 126.5, 122.5 (arom CH), 55.1 (C-4), 36.7 (-C(CH_3_)_3_), 27.5 (-C(CH_3_)_3_). Anal. Calcd for C_19_H_20_N_2_O_2_: C, 74.00; H, 6.54; N, 9.08. Found: C, 74.16; H, 6.55; N, 9.07.

*4-Benzyl-1,2-diphenylpyrazolidine-3,5-dione* (**1g**).Colorless blocks (from CHCl_3_-hexane); mp
136–138 °C (lit, mp 135–137 °C); IR (KBr) ** 1747, 1718 (C=O); ^1^H-NMR (300 MHz, CDCl_3_) *δ*
7.26–6.86 (15H, m, arom H), 3.65 (1H, t, *J* = 4.5 Hz, -CH-), 3.44 (2H, d, *J* = 4.5 Hz, -CH_2_-); ^13^C-NMR (75 MHz, CDCl_3_) *δ* 169.3 (C=O), 135.3, 135.0 (arom C), 129.9, 128.7, 128.6, 127.3, 127.0, 123.2 (arom CH), 48.5 (C-4), 33.9 (-CH_2_-). Anal. Calcd for C_22_H_18_N_2_O_2_: C, 77.17; H, 5.30; N, 8.18. Found: C, 77.16; H, 5.20; N, 8.08.

*4-Cyclopentyl-1,2-diphenylpyrazolidine-3,5-dione* (**1h**): Colorless microcrystals (from CH_2_Cl_2_-hexane); mp 170 °C; IR (KBr) ** 1749, 1720 (C=O); ^1^H-NMR (300 MHz, CDCl_3_) *δ* 7.30–7.12 (10H, m, arom H), 3.41 (1H, d, *J* = 4.5 Hz, -CH-), 2.60–2.59 (1H, m, -(CH_2_)_2_-CH-(CH_2_)_2_-), 1.89–1.55 (8H, m,
-(CH_2_)_4_-); ^13^C-NMR (75 MHz, CDCl_3_) *δ* 170.0 (C=O), 135.7 (arom C), 128.8, 126.6, 122.4 (arom CH), 48.5 (C-4), 40.7 (-(CH_2_)_2_-CH-(CH_2_)_2_-), 28.7, 24.8 (-(CH_2_)_4_-). Anal. Calcd for C_20_H_20_N_2_O_2_: C, 74.98; H, 6.29; N, 8.74. Found: C, 74.94; H, 6.23; N, 8.69.

The physical data for the pyrrolidinediones **3a**–**s** are given below.

*1-Benzyl-4-hydroxy-3-methyl-3-pyrrolin-2-one* (**3a**). *R*_f_ = 0.57 (EtOAc:hexane = 9:1 v/v); colorless needles (from EtOH/hexane); mp 146–150 °C; IR (CHCl_3_) ** 3400–3100 (OH), 1784, 1697 (C=O);  ^1^H-NMR (DMSO-*d*_6_) *δ* 10.65 (1H, s, OH), 7.15–7.35 (5H, m, arom H), 4.46 (2H, s, CH_2_-C=), 3.63 (2H, s, PhCH_2_), 1.58 (3H, s, Me); ^13^C-NMR (DMSO-*d*_6_) *δ* 173.2 (C-2, C=O), 164.8 (C-4), 138.4, 128.6, 127.4, 127.1 (arom C), 100.2 (C-3), 49.0 (C-5, CH_2_), 44.6 (PhCH_2_), 6.4 (Me). FAB HRMS (acetone/NBA) calcd for C_12_H_14_NO_2_ 204.1025 (M+H). Found 204.1002.

*1-Benzyl-3-ethyl-4-hydroxy-3-pyrrolin-2-one* (**3b**).*R*_f_ = 0.61 (EtOAc:hexane = 7:3 v/v); colorless cubes (from EtOH/hexane); mp 117–118 °C; IR (CHCl_3_) ** 3300–3000 (weak OH), 1770, 1693 (C=O); ^1^H-NMR (DMSO-*d*_6_) *δ* 10.70 (1H, br s, OH), 7.16–7.36 (5H, m, arom H), 4.46 (2H, s, CH_2_-C=), 3.64 (2H, s, PhCH_2_), 2.11 (2H, q, *J* = 7.8 Hz, CH_2_), 0.98 (3H, t, *J* = 7.8 Hz, Me); ^13^C-NMR (DMSO-*d*_6_) *δ* 172.7 (C-2, C=O), 164.3 (C-4), 138.3, 128.5, 127.3, 126.9 (arom C), 106.2 (C-3), 48.8 (C-5, CH_2_), 44.5 (PhCH_2_), 14.5 (CH_2_), 13.0 (Me). Anal. Calcd for C_13_H_15_NO_2_•1/3 H_2_O: C, 69.93; H, 7.07; N, 6.27. Found: C, 69.67; H, 6.86; N, 6.30.

*1-Benzyl-4-hydroxy-3-propyl-3-pyrrolin-2-one* (**3c**).*R*_f_ = 0.75 (EtOAc:hexane = 7:3 v/v); colorless needles (from CHCl_3_/hexane); mp 124 °C; IR (CHCl_3_) * *  1768, 1689 (C=O);  ^1^H-NMR (DMSO-*d*_6_) *δ* 10.65 (1H, br s, OH), 7.16–7.36 (5H, m, arom H), 4.47 (2H, s, CH_2_-C=), 3.65 (2H, s, PhCH_2_), 2.07 (2H, t, *J* = 7.5 Hz, CH_2_), 1.41 (2H, m, CH_2_), 0.85 (3H, t, *J* = 7.5 Hz, Me);  ^13^C-NMR (DMSO-*d*_6_) *δ* 172.9 (C-2, C=O), 164.9 (C-4), 138.4, 128.6, 127.4, 127.1 (arom C), 104.8 (C-3), 48.9 (C-5, CH_2_), 44.6 (PhCH_2_), 23.3, 21.3 (CH_2_), 13.9 (Me). FAB HRMS (acetone/NBA)calcd for C_14_H_18_NO_2_ 232.1338 (M+H). Found 232.1340.

*1-Benzyl-3-butyl-4-hydroxy-3-pyrrolin-2-one* (**3d**).*R*_f_ = 0.44 (EtOAc:hexane = 5:5 v/v); colorless needles (from EtOH/H_2_O); mp 101–103 °C; IR (CHCl_3_) ** 3300–3000 (OH), 1770, 1689 (C=O);  ^1^H-NMR (DMSO-*d*_6_) *δ* 10.59 (1H, br s, OH), 7.38–7.16 (5H, m, arom H), 4.47 (2H, s, CH_2_-C=), 3.65 (2H, s, PhCH_2_), 2.11 (2H, t, *J* = 4.5 Hz, CH_2_), 1.40 (2H, m, CH_2_), 1.28 (2H, m, CH_2_), 0.88 (3H, t,  *J* = 4.5 Hz, Me); ^13^C-NMR (DMSO-*d*_6_) *δ* 172.8 (C-2, C=O), 164.7 (C-4), 138.3, 128.5, 127.1, 126.9 (arom C), 104.8 (C-3), 48.8 (C-5, CH_2_), 44.8 (PhCH_2_), 30.1, 21.9, 20.8 (CH_2_), 13.7 (Me). FAB HRMS (acetone/NBA) calcd for C_15_H_20_NO_2_ 246.1494 (M+H). Found 246.1487.

*1-Benzyl-3-hexyl-4-hydroxy-3-pyrrolin-2-one* (**3e**).*R*_f_ = 0.62 (EtOAc:hexane = 5:5 v/v); colorless needles (from EtOH/hexane); mp 94–95 °C; IR (CHCl_3_) ** 3600–3300 (weak OH), 1768, 1689 (C=O); ^1^H-NMR (DMSO-*d*_6_) *δ* 10.68 (1H, br s, OH), 7.19–7.39 (5H, m, arom H), 4.50 (2H, s, CH_2_-C=), 3.68 (2H, s, PhCH_2_), 2.13 (2H, t, *J* = 9.0 Hz, CH_2_), 1.29–1.46 (8H, m, 4CH_2_), 0.89 (3H, t, *J* = 9.0 Hz, Me); ^13^C-NMR (DMSO-*d*_6_) *δ* 173.0 (C-2, C=O), 164.8 (C-4), 138.4, 128.6, 127.4, 127.1 (arom C), 105.0 (C-3), 48.6 (C-5, CH_2_), 44.6 (PhCH_2_), 31.2, 28.6, 27.9, 22.2, 21.2 (CH_2_), 13.9 (Me). FAB HRMS (acetone/NBA) calcd for C_17_H_24_NO_2_ 274.1807 (M+H). Found 274.1795.

*1-Butyl-4-hydroxy-3-methyl-3-pyrrolin-2-one* (**3f**).*R*_f_ = 0.32 (CHCl_3_:MeOH = 98:2 v/v); colorless needles (from ethanol/hexane); mp 110–112 °C; IR (CHCl_3_) ** 3250–3000 (weak OH), 1772, 1685 (C=O); ^1^H-NMR (DMSO-*d*_6_) *δ* 10.56 (1H, br s, OH), 3.71 (2H, s, CH_2_-C=), 3.23 (2H, t, *J* = 6.9 Hz, CH_2_), 1.53(3H, s, Me), 1.43 (2H, m, CH_2_), 1.24 (2H, m, CH_2_), 0.87 (3H, t, *J* = 7.2 Hz, Me); ^13^C-NMR (DMSO-*d*_6_) *δ* 172.9 (C-2, C=O), 164.2 (C-4), 100.3 (C-3), 49.2 (C-5, CH_2_), 44.6 (PhCH_2_), 40.3, 30.2, 19.5 (CH_2_), 13.7 (Me), 6.4 (Me). FAB HRMS (acetone/NBA) calcd for C_9_H_16_NO_2_ 170.1181 (M+H). Found 170.1178.

*1-Butyl-3-ethyl-4-hydroxy-3-pyrrolin-2-one* (**3g**).*R*_f_ = 0.49 (EtOAc:hexane = 6:4 v/v); colorless needles (from EtOAc/hexane); mp 85–87 °C; IR (CHCl_3_) ** 3500–3100 (OH), 1768, 1685 (C=O);
^1^H-NMR (DMSO-*d*_6_) *δ* 10.47 (1H, br s, OH), 3.70 (2H, s, CH_2_-C=), 3.23 (2H, t, *J* = 6.9 Hz, CH_2_), 2.04 (2H, q, *J* = 7.2 Hz, CH_2_), 1.43 (2H, m, CH_2_), 1.21 (2H, m, CH_2_), 0.89 (6H, t, *J* = 7.2 Hz, 2Me);  ^13^C-NMR (DMSO-*d*_6_) *δ* 172.4 (C-2, C=O), 163.8 (C-4), 106.4 (C-3), 48.9 (C-5, CH_2_), 40.3, 30.1, 19.4, 14.4 (CH_2_), 13.7 (Me), 6.4 (Me). FAB HRMS (acetone/NBA) calcd for C_10_H_18_NO_2_ 184.1338 (M+H). Found 184.1338.

*1,3-Dibutyl-4-hydroxy-3-pyrrolin-2-one* (**3h**). *R*_f_ = 0.69 (EtOAc:hexane = 6:4 v/v); colorless microcrystals (from cold water); mp 113–114 °C; IR (CHCl_3_) ** 3300–3100 (OH), 1770, 1693 (C=O); ^1^H-NMR (DMSO-*d*_6_) *δ* 10.59 (1H, br s, OH), 3.77 (2H, s, CH_2_-C=), 3.29 (2H, t, *J* = 6.9 Hz, CH_2_), 2.09 (2H, t, *J* = 7.5 Hz, CH_2_), 1.29–1.49 (8H, m, 4CH_2_), 0.93 (6H, t, *J* = 7.2 Hz, 2Me); ^13^C-NMR (DMSO-*d*_6_) *δ* 172.8 (C-2, C=O), 164.5 (C-4), 105.3 (C-3), 49.1 (C-5, CH_2_), 40.3, 30.1, 21.9, 20.7, 19.4 (CH_2_), 13.7 (Me), 13.5 (Me). FAB HRMS (acetone-NBA) calcd for C_12_H_22_NO_2_ 212.1651 (M+H). Found 212.1635.

*1-(*t*-Butyl)-4-hydroxy-3-methyl-3-pyrrolin-2-one* (**3i**).*R*_f_ = 0.72 (EtOAc:hexane = 8:2 v/v); colorless needles (from EtOAc/hexane); mp 184 °C; IR (CHCl_3_) ** 3300–3000 (weak OH), 1768, 1689 (C=O); ^1^H-NMR (DMSO-*d*_6_) *δ* 10.36 (1H, br s, OH), 3.76 (2H, s, CH_2_-C=), 1.48 (3H, s, Me), 1.32 (9H, s, 3Me); ^13^C-NMR (DMSO-d_6_) *δ* 173.3 (C-2, C=O), 163.4 (C-4), 101.6 (C-3), 52.3 (quart C), 47.5 (C-5, CH_2_), 27.7 (3Me), 6.2 (Me). FAB HRMS (acetone-NBA) calcd for C_9_H_16_NO_2_ 170.1181 (M+H). Found 170.1179.

*1-(*t*-Butyl)-3-ethyl-4-hydroxy-3-pyrrolin-2-one* (**3j**).*R*_f_ = 0.32 (EtOAc:hexane = 4:6 v/v); colorless needles (from CHCl_3_/hexane); mp 129–130 °C; IR (CHCl_3_) ** 3300-3100 (weak OH), 1768, 1683 (C=O); ^1^H-NMR (DMSO-*d*_6_) *δ* 10.37 (1H, br s, OH), 3.76 (2H, s, CH_2_-C=), 2.00 (2H, q, *J* = 7.5 Hz, CH_2_), 1.32 (9H, s, 3Me), 0.92 (3H, t, *J* = 7.5 Hz, Me); ^13^C-NMR (DMSO-*d*_6_) *δ* 172.9 (C-2, C=O), 163.1 (C-4), 107.6 (C-3), 52.3 (quart C), 47.4 (C-5, CH_2_), 27.7 (3Me), 14.3 (CH_2_), 13.1 (Me). FAB HRMS (acetone/NBA) calcd for C_10_H_18_NO_2_ 184.1338 (M+H). Found 184.1325.

*1-(*t*-Butyl)-4-hydroxy-3-propyl-3-pyrrolin-2-one* (**3k**).*R*_f_ = 0.64 (EtOAc:hexane = 5:5 v/v); colorless needles (from EtOH/hexane); mp 145–146 °C; IR (CHCl_3_) ** 3400-3100 (weak OH), 1766, 1687 (C=O); ^1^H-NMR (DMSO-*d*_6_) *δ* 10.31 (1H, br s, OH), 3.77 (2H, s, CH_2_-C=), 1.97 (2H, t, *J* = 7.8 Hz, CH_2_), 1.38 (2H, m, CH_2_), 1.36 (9H, s, 3Me), 0.82 (3H, t, *J* = 7.2 Hz, Me); ^13^C-NMR (DMSO-*d*_6_) *δ* 173.1 (C-2, C=O), 163.6 (C-4), 106.2 (C-3), 52.3 (quart C), 47.4 (C-5, CH_2_), 27.7 (3Me), 23.1, 21.2 (CH_2_), 13.9 (Me). FAB HRMS (acetone/NBA) calcd for C_11_H_20_NO_2_ 198.1494 (M+H). Found 198.1487.

*1-(*t*-Butyl)-3-butyl-4-hydroxy-3-pyrrolin-2-one* (**3l**).*R*_f_ = 0.59 (EtOAc:hexane = 5:5 v/v); colorless needles (from EtOH/hexane); mp 142–144 °C; IR (CHCl_3_) ** 3500–3050 (OH), 1766, 1687 (C=O);
^1^H-NMR (DMSO-*d*_6_) *δ* 10.33 (1H, br s, OH), 3.76 (2H, s, CH_2_-C=), 1.99 (2H, t, *J* = 4.2 Hz, CH_2_), 1.29 (9H, s, 3Me), 1.23 (4H, m, 2CH_2_), 0.82 (3H, t, *J* = 7.2 Hz, Me); ^13^C-NMR (DMSO-*d*_6_) *δ* 173.2 (C-2, C=O), 163.6 (C-4), 106.4 (C-3), 52.4 (quart C), 47.5 (C-5, CH_2_), 27.8 (3Me), 30.1, 22.1, 20.8 (CH_2_), 13.8 (Me). FAB HRMS (acetone/NBA) calcd for C_12_H_22_NO_2_ 212.1651 (M+H). Found 212.1637.

*1-(*t*-Butyl)-3-hexyl-4-hydroxy-3-pyrrolin-2-one* (**3m**).*R*_f_ = 0.53 (EtOAc:hexane = 3:7 v/v); colorless needles (from EtOH/hexane); mp 94 °C; IR (CHCl_3_) ** 3300–3100 (weak OH), 1780, 1683 (C=O);  ^1^H-NMR (DMSO-*d*_6_) *δ* 10.32 (1H, br s, OH), 3.74 (2H, s, CH_2_-C=), 1.95 (2H, t, *J* = 7.8 Hz, CH_2_), 1.36 (9H, s, 3Me), 1.36–1.21 (8H, m, 4CH_2_), 0.83 (3H, t, *J* = 7.2 Hz, Me); ^13^C-NMR (DMSO-*d*_6_) * *173.2 (C-2, C=O), 163.5 (C-4), 106.4 (C-3), 52.4 (quart C), 47.4 (C-5, CH_2_), 31.1, 28.7 (CH_2_), 27.9 (3Me), 27.8, 22.1, 21.0 (CH_2_), 13.9 (Me). FAB HRMS (acetone/NBA) calcd for C_14_H_26_NO_2_ 240.1964 (M+H). Found 240.1948.

*4-Hydroxy-3-methyl-1-(*i*-propyl)-3-pyrrolin-2-one* (**3n**).*R*_f_ = 0.30 (EtOAc:hexane = 7:3 v/v); colorless needles (from EtOAc/hexane); mp 140–142 °C; IR (CHCl_3_) ** 3300–3100 (weak OH), 1770, 1683 (C=O); ^1^H-NMR (DMSO-*d*_6_) *δ* 10.56 (1H, br s, OH), 4.15 (1H, sep, *J* = 6.9 Hz, -CH<), 3.66 (2H, s, CH_2_-C=), 1.52 (3H, s, Me), 1.06 (6H, d, *J* = 6.9 Hz, 2Me); ^13^C-NMR (DMSO-*d*_6_) *δ* 72.3 (C-2, C=O), 164.4 (C-4), 100.5 (C-3), 44.5 (C-5, CH_2_), 41.1 (CH), 20.3 (2Me), 6.2 (Me). FAB HRMS (acetone/NBA) calcd for C_8_H_14_NO_2_ 156.1025 (M+H). Found 156.1023.

*3-Ethyl-4-hydroxy-1-(*i*-propyl)-3-pyrrolin-2-one* (**3o**):*R*_f_ = 0.55 (EtOAc:hexane = 7:3 v/v); colorless needles (from EtOAc/hexane); mp 117–118 °C; IR (CHCl_3_) ** 3300–3100 (weak OH), 1770, 1683 (C=O); ^1^H-NMR (DMSO-*d*_6_) *δ* 10.52 (1H, br s, OH), 4.15 (1H, sep, *J* = 6.0 Hz, -CH<), 3.65 (2H, s, CH_2_-C=), 2.04 (2H, q, *J* = 6.0 Hz, CH_2_), 1.06 (6H, d, *J* = 6.0 Hz, 2Me), 0.94 (3H, t, *J* = 6.0 Hz, Me); ^13^C-NMR (DMSO-*d*_6_) *δ* 172.0 (C-2, C=O), 164.0 (C-4), 106.7 (C-3), 44.5 (C-5, CH_2_), 41.0 (CH), 20.4 (2Me), 14.4 (CH_2_), 13.1 (Me). FAB HRMS (acetone/NBA) calcd for C_9_H_16_NO_2_ 170.1181 (M+H). Found 170.1171.

*4-Hydroxy-1-(*i*-propyl)-3-propyl-3-pyrrolin-2-one* (**3p**). *R*_f_ = 0.55 (EtOAc:hexane = 7:3 v/v); colorless needles (from EtOAc/hexane); mp 117–118 °C; IR (CHCl_3_) ** 3300–3100 (OH), 1770, 1681 (C=O); ^1^H-NMR (DMSO-*d*_6_) *δ* 10.49 (1H, br s, OH), 4.16 (1H, sep, *J* = 6.9 Hz, -CH<), 3.66 (2H, s, CH_2_-C=), 2.00 (2H, t, *J* = 7.8 Hz, CH_2_), 1.36 (2H, m, CH_2_), 1.07 (6H, d, *J* = 6.9 Hz, 2Me), 0.83 (3H, t, *J* = 7.8 Hz, Me); ^13^C-NMR (DMSO-*d*_6_) *δ* 172.2 (C-2, C=O), 164.6 (C-4), 105.2 (C-3), 44.5 (C-5, CH_2_), 41.1 (CH), 23.0, 21.2 (CH_2_), 20.3 (2Me), 13.8 (Me). FAB HRMS (acetone/NBA) calcd for C_10_H_18_NO_2_ 184.1338 (M+H). Found 184.1334.

*1-Ethyl-4-hydroxy-3-methyl-3-pyrrolin-2-one* (**3q**):*R*_f_ = 0.32 (EtOAc:hexane = 6:4 v/v); colorless microcrystals (from EtOAc/hexane); mp 121–122 °C; IR (CHCl_3_) ** 3300–3100 (OH), 1772, 1685 (C=O); ^1^H-NMR (DMSO-*d*_6_) *δ* 10.54 (1H, br s, OH), 3.72 (2H, s, CH_2_-C=), 3.27 (2H, q, *J* = 7.2 Hz, CH_2_), 1.53 (3H, s, Me), 1.02 (3H, t, *J* = 7.2 Hz, Me); ^13^C-NMR (DMSO-*d*_6_) *δ* 172.6 (C-2, C=O), 164.3 (C-4), 100.4 (C-3), 48.6 (C-5, CH_2_), 35.4 ( CH_2_), 15.5, 6.2 (Me). FAB HRMS (acetone/NBA)calcd for C_7_H_11_NO_2_Na 164.0687 (M+Na). Found 164.0692.

*Ethyl 1-benzyl-4-hydroxy-3-pyrrolin-2-one-3-carboxylate* (**3r**).*R*_f_ = 0.68 (CHCl_3_:MeOH = 98:2 v/v); colorless microcrystals (from benzene/cyclohexane); mp 148–150 °C (lit., mp 148.5–150 °C); IR (CHCl_3_) ** 3700–3200 (OH), 1706, 1602 (C=O); ^1^H-NMR (CDCl_3_) *δ* 7.36–7.23 (5H, m, arom H), 4.59 (2H, s, CH_2_), 4.41 (2H, q, *J* = 7.2 Hz, CH_2_), 3.85 (2H, s, CH_2_-C=), 1.63 (1H, br s, OH), 1.41 (3H, t,  *J* = 7.2 Hz, Me); ^13^C-NMR (CDCl_3_) *δ* 183.8, 167.1 (C=O), 157.6 (C-4), 136.6 (arom C), 128.8, 128.1, 127.8 (arom CH), 98.6 (C-3), 61.3, 49.0, 45.4 (CH_2_), 14.2 (Me).

*1-Benzyl-4-hydroxy-3-phenyl-3-pyrrolin-2-one* (**3s**).*R*_f_ = 0.29 (CHCl_3_:MeOH = 98:2 v/v); colorless needles (from EtOH/hexane); mp 197–198 °C (lit., mp 197–198 °C); IR (KBr) ** 3500-2900 (OH), 1664 (C=O); ^1^H-NMR (DMSO-*d*_6_) *δ* 11.71 (1H, br s, OH), 8.02–7.14 (10H, m, arom H), 4.56 (2H, s, CH_2_), 3.84 (2H, s, CH_2_); ^13^C-NMR (DMSO-*d*_6_) *δ* 170.9 (C-2, C=O), 166.9 (C-4), 138.0, 132.2, 128.5 (arom C), 127.6, 127.4, 127.0, 126.6, 125.5 (arom CH), 102.7 (C-3), 48.6 (C-5, CH_2_), 44.4 (CH_2_).

The physical data for the barbituric acids **5a**–**e** are given below.

*1,3-Dimethyl-5-*i*-proprylbarbituric acid* (**5a**). Yield 90%; colorless microcrystals (from CH_2_Cl_2_/hexane); mp 105 °C; IR (CHCl_3_) ** 1739, 1697, 1667 (C=O); ^1^H-NMR (300 MHz, CDCl_3_) *δ* 3.34 (1H, d, *J* = 3.8 Hz, -CH- of C-5), 3.29 (6H, s, 2 × CH_3_), 2.60–2.54 (1H, m, CH_3_-CH-CH_3_), 1.06 (6H, d, *J* = 6.8 Hz,
CH_3_-CH-CH_3_); ^13^C-NMR (75 MHz, CDCl_3_) *δ* 168.3, 151.7 (C=O), 55.2 (C-5), 36.8 (CH_3_-CH-CH_3_,
*i*-Pr), 28.1 (CH_3_), 19.4 (CH_3_-CH-CH_3_, *i*-Pr). Anal. Calcd for C_9_H_14_N_2_O_3_: C, 54.53; H, 7.12; N, 14.13. Found: C, 54.60; H, 7.06; N, 14.11.

*5-Benzyl-1,3-dimethylbarbituric acid* (**5b**). Colorless blocks (from CH_2_Cl_2_/hexane); mp 112 °C; IR (KBr) ** 1743, 1697, 1678 (C=O); ^1^H-NMR (300 MHz, CDCl_3_) *δ* 7.23–7.00 (5H, m, arom H),
3.78–3.75 (1H, m, -CH-), 3.44 (2H, d, *J* = 4.6 Hz, -CH_2_-, Bn), 3.10 (6H, s, 2 × CH_3_); ^13^C-NMR (75 MHz, CDCl_3_) *δ* 168.3, 150.9 (C=O), 135.1 (arom C), 128.8, 128.5, 127.7 (arom CH), 50.6 (C-5), 37.8 (-CH_2_-, Bn), 28.1 (CH_3_). Anal. Calcd for C_13_H_14_N_2_O_3_•2/3H_2_O: C, 60.46; H, 5.98; N, 10.85. Found: C, 60.54; H, 5.48; N, 12.22.

*5-(4-Methoxyphenyl)methyl-1,3-dimethylbarbituric acid* (**5c**). Colorless blocks (from CH_2_Cl_2_/hexane); mp 85 °C; IR (KBr) ** 1743, 1698, 1660 (C=O); ^1^H-NMR (300 MHz, CDCl_3_) *δ* 6.94–6.71 (4H, m, arom H), 3.73 (3H, s, CH_3_O-), 3.71–3.67 (1H, m, -CH- of C-5), 3.38–3.36 (2H, m, -CH_2_-), 3.10 (3H, s, CH_3_), 3.04 (3H, s, CH_3_); ^13^C-NMR (75 MHz, CDCl_3_) *δ* 168.1, 158.7, 150.7 (C=O), 126.8 (arom C), 129.7, 113.6 (arom CH), 54.8 (CH_3_O), 50.5 (C-5), 36.7 (-CH_2_-), 27.8 (CH_3_). Anal. Calcd for C_14_H_16_N_2_O_4_: C, 60.86; H, 5.84; N, 10.14. Found: C, 60.58; H, 5.43; N, 10.15.

*5-(2-Methoxyphenyl)methyl-1,3-dimethylbarbituric acid* (**5d**). Pale yellow needles (from CH_2_Cl_2_/hexane); mp 107 °C (decompd); IR (KBr) ** 1745, 1701, 1674 (C=O); ^1^H-NMR (300 MHz, CDCl_3_) *δ* 7.23–6.78 (4H, m, arom H), 3.80-3.77 (1H, m, -CH- of C-5), 3.73 (3H, s, CH_3_O), 3.40 (2H, d, *J* = 5.4 Hz, -CH_2_-), 3.10 (6H, s, 2 × CH_3_); ^13^C-NMR (75 MHz, CDCl_3_) *δ* 168.3, 157.1, 151.4 (C=O), 123.3 (arom C), 131.0, 123.3, 120.3, 110.0 (arom CH), , 55.0 (CH_3_O), 50.0 (C-5), 33.3 (-CH_2_-), 28.0 (CH_3_). Anal. Calcd for C_14_H_16_N_2_O_4_: C, 60.86; H, 5.84; N, 10.14. Found: C, 60.86; H, 5.85; N, 10.07.

*5-(2-Naphthyl)methyl-1,3-Dimethylbarbituric acid*(**5e**). Yield 88%; colorless microcrystals (from CH_2_Cl_2_/hexane); mp 130 °C; IR (KBr) ** 1693, 1670 (C=O); ^1^H-NMR (300 MHz, CDCl_3_) *δ* 7.78–7.12 (7H, m, arom H), 3.84 (1H, t, *J* = 4.8 Hz, -CH- of C-5), 3.63 (2H, d, *J* = 4.8 Hz, -CH_2_-), 3.09 (3H, s, CH_3_), 3.01 (3H, s, CH_3_); ^13^C-NMR (75 MHz, CDCl_3_) *δ* 168.2 (C=O), 133.3, 132.7, 132.6 (arom C), 128.3, 127.9, 127.7, 127.5, 126.7, 126.4, 126.0 (arom CH), 50.7 (C-5), 37.5 (-CH_2_-), 28.2 (CH_3_).

The physical data for the quinolinone **7** are given below.

*3-Butyl-4-hydroxy-2-quinolinone* (**7**). Yellow microcrystals (from EtOAc/hexane); mp 196 °C (lit., mp 199–200 °C); IR (KBr) ** 3400–3000 (OH and NH), 1639 (C=O); ^1^H-NMR (300 MHz, CDCl_3_) *δ* 10.75 (1H, s, NH), 7.32–6.52 (4H, m, arom H), 2.01–1.97 (2H, m, -CH_2_-C_3_H_7_), 0.86–0.73 (4H, m, -CH_2_-CH_2_-CH_2_-CH_3_), 0.30 (3H, t, *J* = 7.2 Hz, -C_3_H_6_-CH_3_); ^13^C-NMR (75 MHz, CDCl_3_) *δ* 163.5 (C=O), 156.8 (C-4), 137.2, 115.3 (arom C), 129.4, 122.4, 120.7, 114.6 (arom CH), 111.7 (C-3), 30.3 (-CH_2_-C_3_H_7_), 22.7 (-CH_2_-CH_2_-CH_2_-CH_3_), 22.2 (-CH_2_-CH_2_-CH_2_-CH_3_), 13.9 (-C_3_H_6_-CH_3_).

The physical data for the malonamides **11a** and **11b** are given below.

*2-Benzyl-N^1^,N^3^-dipropylmalonamide* (**11a**). 1.19 g (43% yield); *R*_f_ = 0.09 (CHCl_3_); colorless microcrystals (from CHCl_3_/hexane); mp 106–107 °C; IR (KBr) ** 3500–3200 (NH), 1630 (C=O);  ^1^H-NMR (300 MHz, CDCl_3_) *δ* 7.28–7.16 (5H, m), 6.88 (2H, br. s), 3.24–3.08 (6H, m), 1.42 (4H sex,  *J* = 7.2 Hz), 0.82 (6H, t, *J* = 7.2 Hz); ^13^C-NMR (75 MHz, CDCl_3_) *δ* 170.4, 138.1, 128.8, 128.6, 126.8, 57.3, 41.3, 39.0, 22.5, 11.3.

*2-Benzyl-N^1^,N^3^-dibutylmalonamide* (**11b**). 2.62 g (86% yield); *R*_f_ = 0.1 (CHCl_3_); colorless needles (from CHCl_3_/hexane); mp 108–109 °C; IR (KBr) ** 3400–3200 (NH), 1659 (C=O); ^1^H-NMR (300 MHz, CDCl_3_) *δ* 7.28–7.16 (5H, m), 6.81 (2H, br. s), 3.19–3.12 (6H, m), 1.37 (4H, quin, *J* = 7.2 Hz), 1.23 (4H, sex, *J* = 7.2 Hz), 0.87 (6H, t, *J* = 7.2 Hz); ^13^C-NMR (75 MHz, CDCl_3_)* * 170.3, 138.1, 128.8, 128.5, 126.8, 57.2, 39.3, 39.0, 31.3, 19.9, 13.7. Anal. Calcd for C_18_H_28_N_2_O_2_: C, 71.02; H, 9.27; N, 9.20. Found: C, 70.84; H, 9.50; N, 9.26.

The physical data for the hydroperoxides **2a**–**h**, **4a**–**p**, **6a**–**e**, and **8** are given below.

*4-Hydroperoxy-4-methyl-1,2-diphenylpyrazolidine-3,5-dione* (**2a**). Colorless microcrystals (from CH_2_Cl_2_/hexane); mp 156–158 °C (decompd); IR (KBr) ** 3500–3200 (OOH), 1762, 1724 (C=O);  ^1^H-NMR (300MHz, CDCl_3_) *δ* 11.06 (1H, s, OOH), 7.34–7.16 (10H, m, arom H), 1.60 (3H, s, CH_3_); ^13^C-NMR (75 MHz, CDCl_3_) *δ* 168.9 (C=O), 134.9 (arom C), 129.1, 127.6, 123.4 (arom CH), 81.6 (>C-O), 17.7 (CH_3_). Anal. Calcd for C_16_H_14_N_2_O_4_: C, 64.42; H, 4.73; N, 9.39. Found: C, 64.19; H, 4.70; N, 9.28.

*4-Ethyl-4-hydroperoxy-1,2-diphenylpyrazolidine-3,5-dione* (**2b**). Colorless microcrystals (from EtOAc/exane); mp 141 °C; IR (KBr) ** 3600–3100 (OOH), 1762, 1724 (C=O); ^1^H-NMR (300 MHz, CDCl_3_) *δ* 10.67 (1H, s, OOH), 7.34–7.18 (10H, m, arom H), 2.04 (2H, q, *J* = 7.5 Hz, -CH_2_-CH_3_, Et), 1.03 (3H, t, *J* = 7.5 Hz, -CH_2_-CH_3_, Et); ^13^C-NMR (75 MHz, CDCl_3_) *δ* 168.5 (C=O), 134.9 (arom C), 129.1, 127.4, 123.2 (arom CH), 85.8 (>C-O), 25.8 (-CH_2_-CH_3_, Et), 6.8 (-CH_2_-CH_3_, Et). Anal. Calcd for C_17_H_16_N_2_O_4_: C, 65.38; H, 5.16; N, 8.97. Found: C, 65.10; H, 5.13; N, 8.98.

*4-Hydrperoxy-4-propyl-1,2-diphenylpyrazolidine-3,5-dione*(**2c**). Colorless microcrystals (from Et_2_O/hexane); mp 137 °C; IR (KBr) ** 3550–3200 (OH), 1764, 1726 (C=O); ^1^H-NMR (300 MHz, CDCl_3_) *δ* 10.95 (1H, s, OOH), 7.34–7.17 (10H, m, arom H), 1.98–1.93 (2H, m, -CH_2_-C_2_H_5_), 1.47-1.41 (2H, m, -CH_2_-CH_2_-CH_3_), 0.94 (3H, t, *J* = 7.2 Hz, -CH_2_-CH_2_-CH_3_); ^13^C-NMR (75 MHz, CDCl_3_) *δ* 168.8 (C=O), 134.9 (arom C), 129.1, 127.5, 123.3 (arom CH), 85.3 (>C-O), 34.3 (-CH_2_-CH_2_-CH_3_), 16.0 (-CH_2_-CH_2_-CH_3_), 14.0 (-CH_2_-CH_2_-CH_3_). Anal. Calcd. for C_18_H_18_N_2_O_4_: C, 66.25; H, 5.56; N, 8.54. Found: C, 65.99; H, 5.49; N, 8.45.

*4-Hydroperoxy-4-*i*-propyl-1,2-diphenylpyrazolidine-3,5-dione*(**2d**). Colorless microcrystals (from Et_2_O/hexane); mp 149–151 °C; IR (KBr) ** 3500–3150 (OOH), 1761, 1720 (C=O); ^1^H-NMR (300 MHz, CDCl_3_) *δ* 10.71 (1H, s, OOH), 7.37–7.16 (10H, m, arom H), 2.44 (1H, sep, *J* = 6.9 Hz, CH_3_-CH-CH_3_), 1.12 (6H, d, *J* = 6.9 Hz, CH_3_-CH-CH_3_); ^13^C-NMR (75 MHz, CDCl_3_) *δ* 168.6 (C=O), 135.0 (arom C), 129.0, 127.4, 123.1 (arom CH), 87.6 (>C-O), 33.4 (CH_3_-CH-CH_3_), 15.9 (CH_3_-CH-CH_3_). Anal. Calcd for C_18_H_18_N_2_O_4_: C, 66.25; H, 5.56; N, 8.58. Found: C, 65.97; H, 5.50; N, 8.49.

*4-Butyl-4-hydroperoxy-1,2-diphenylpyrazolidine-3,5-dione* (**2e**). Colorless microcrystals (from EtOAc/hexane); mp 124–126 °C (decompd), (lit, [7] mp 110–111 °C); IR (KBr) ** 3500–3150 (OOH), 1763, 1724 (C=O); ^1^H-NMR (300 MHz, CDCl_3_) *δ* 10.72 (1H, s, OOH), 7.34–7.18 (10H, m, arom H), 2.01–1.95 (2H, m, -CH_2_-C_3_H_7_), 1.40–1.31 (4H, m, -CH_2_-CH_2_-CH_2_-CH_3_), 0.85 (3H, t, *J* = 6.9 Hz,
-CH_2_-CH_2_-CH_2_-CH_3_); ^13^C-NMR (75 MHz, CDCl_3_) *δ* 168.7 (C=O), 134.9 (arom C), 129.0, 127.4, 123.2 (arom CH), 85.2 (>C-O), 32.0 (-CH_2_-CH_2_-CH_2_-CH_3_), 24.3 (-CH_2_-CH_2_-CH_2_-CH_3_), 22.5 (-CH_2_-CH_2_-CH_2_-CH_3_), 13.5 (-CH_2_-CH_2_-CH_2_-CH_3_). Anal. Calcd for C_19_H_20_N_2_O_4_: C, 67.05; H, 5.92; N, 8.23. Found: C, 66.83; H, 5.67; N, 8.05.

*4-*t*-Butyl-4-hydroperoxy-1,2-diphenylpyrazolidine-3,5-dione* (**2f**). Colorless needles (from EtOAc/hexane); mp 173–178 °C (decompd); IR (KBr) ** 3500–3150 (OOH), 1747, 1717 (C=O);
^1^H-NMR (300 MHz, CDCl_3_) *δ* 10.51 (1H, s, OOH), 7.34–7.15 (10H, m, arom H), 1.19 (9H, s, 3 × CH_3_); ^13^C-NMR (75 MHz, CDCl_3_) *δ* 168.6 (C=O), 135.2 (arom C), 129.0, 127.2, 123.0 (arom CH), 89.1 (>C-O), 38.7 ((CH_3_)_3_C-), 24.3 ((CH_3_)_3_C-). Anal. Calcd for C_19_H_20_N_2_O_4_: C, 67.05; H, 5.92; N, 8.23. Found: C, 67.07; H, 5.86; N, 8.15.

*4-Benzyl-4-hydroperoxy-1,2-diphenylpyrazolidine-3,5-dione* (**2g**). Colorless blocks (from CH_2_Cl_2_/benzene); mp 103–107 °C (decompd); IR (KBr) ** 3400–3150 (OOH), 1753, 1705 (C=O);  ^1^H-NMR (300MHz, CDCl_3_) *δ* 11.21 (1H, s, OOH), 7.24–6.80 (15H, m, arom H), 3.31 (2H, s, CH_2_); ^13^C-NMR (75 MHz, CDCl_3_) *δ* 167.7 (C=O), 134.0, 130.3 (arom C), 130.6, 128.8, 127.9, 127.6, 123.9 (arom CH), 86.7 (C-O), 38.0 (CH_2_). Anal. Calcd for C_22_H_18_N_2_O_4_•2/3H_2_O: C, 68.38; H, 4.69; N, 7.24. Found: C, 68.13; H, 4.55; N, 7.11. FAB HRMS (acetone/NBA) calcd for C_22_H_19_N_2_O_4_ 375.1345 (M+H). Found 375.1344.

*X-ray crystallographic data of* **2g** (R = Bn)*.* Empirical formula C_22_H_18_N_2_O_4_; formulaweight 374.3894; colorless plates; crystal dimensions 0.25× 0.50 × 0.10 mm; triclinic; space group *P*-1; *a* = 10.3233(5), *b* = 10.4273(4), *c* = 12.9985(6) Å, *α* = 95.792(1)°, *β* = 104.504(3)^o^, *γ* = 105.643(2)°, *V* = 1283.0(1) Å^3^, Z = 2; *D*_calcd_ = 1.272 g/cm^3^; *F*_000_ = 518.00; *μ* (MoK*α*) = 0.85 cm^−1^; 2*θ*_max_ = 55.0°; no. of reflections measured 11910; no. of observations (I > 3.00*σ* (I), 2*θ* < 54.96°) 4605; no. of variables 442; reflection/parameter ratio 10.42; *R* = 0.038; *R_w_* = 0.058; GOF 1.14. The crystallographic data (excluding structure factors) for the structure in this paper have been deposited with the Cambridge Crystallographic Data Centre as supplementary publication number CCDC216767. Copies of the data can be obtained, free of charge, on application to CCDC, 12 Union Road, Cambridge, CB2 1EZ, UK [fax: +44(0)-1223-336033 or e-mail: deposit@ccdc.cam.ac.uk].

*4-Cyclopentyl-4-hydroperoxy-1,2-diphenylpyrazolidine-3,5-dione* (**2h**). Colorless microcrystals (Et_2_O/hexane); mp 189 °C; IR (KBr) ** 3600–3200 (OOH), 1759, 1720 (C=O); ^1^H-NMR (300 MHz, CDCl_3_) *δ* 10.00 (1H, s, OOH), 7.35–7.16 (10H, m, arom H), 2.58–2.46 (1H, quin, *J* = 8.1 Hz, CH), 1.80–1.51 (8H, m, -(CH_2_)_4_); ^13^C-NMR (75 MHz, CDCl_3_) *δ* 168.7 (C=O), 142.9, 135.1(arom C), 129.1, 127.3, 123.1 (arom CH), 86.8 (>C-O), 43.5 (-(CH_2_)_2_-CH-(CH_2_)_2_-), 26.0, 24.8 (-(CH_2_)_4_-). Anal. Calcd for C_34_H_32_N_2_O_4_: C, 68.17; H, 5.72; N, 7.95. Found: C, 68.28; H, 5.72; N, 7.91.

*1-Benzyl-3-hydroperoxy-3-methylpyrrolidine-2,4-dione* (**4a**).Yield (221.1 mg, 94%); *R*_f_ = 0.67 (EtOAc:hexane = 8:2 v/v); colorless blocks (from Et_2_O/hexane); mp 78–79 °C; IR (CHCl_3_) **
3400–3000 (OOH), 1786, 1666 (C=O);^1^H-NMR (CDCl_3_) *δ* 11.28 (1H, s, OOH), 7.36–7.27 (5H, m, arom H), 4.79 ( 1H, d, *J* = 14.7 Hz, CH_2_), 4.64 (1H, d, *J* = 14.7 Hz, CH_2_), 3.75 (2H, s, CH_2_), 1.39(3H, s, Me); ^13^C-NMR (CDCl_3_) *δ* 204.1 (C-4, C=O), 171.3 (C-2, C=O), 134.0, 129.1, 128.4, 128.3 (arom C), 82.5 (C-3), 53.4 (C-5, CH_2_), 46.8 (PhCH_2_), 16.7 (Me). FAB HRMS (acetone/NBA) calcd for C_12_H_14_NO_4_ 236.0923 (M+H). Found 236.0935.

*1-Benzyl-3-ethyl-3-hydroperoxypyrrolidine-2,4-dione* (**4b**).Yield (236.8 mg, 95%); *R*_f_ = 0.53 (EtOAc: hexane = 6:4 v/v); colorless solid; mp 72–76 °C; IR (CHCl_3_) ** 3500–3100 (OOH), 1782, 1693 (C=O); ^1^H-NMR (CDCl_3_) *δ* 11.45 (1H, s, OOH), 7.38–7.26 (5H, m, arom H), 4.73 (2H, s, CH_2_), 3.74 (1H, d,  *J* = 17.7 Hz, CH_2_), 3.64 (1H, d, *J* = 17.7 Hz, CH_2_), 1.85 (2H, q, *J* = 7.4 Hz, CH_2_), 0.89 (3H, t, *J* = 7.4 Hz, Me); ^13^C-NMR (CDCl_3_) *δ* 204.7 (C-4, C=O), 171.0 (C-2, C=O), 134.2, 129.1, 128.4, (arom C), 86.1 (C-3), 54.1 (C-5, CH_2_), 46.8 (PhCH_2_), 25.1 (CH_2_), 16.70 (Me). FAB HRMS (acetone/NBA) calcd for C_13_H_15_NO_4_Na 272.0899 (M+Na). Found 272.0906.

*1-Benzyl-3-hydroperoxy-3-propylpyrrolidine-2,4-dione* (**4c**).Yield (258.0 mg, 98%); *R*_f_ = 0.54 (EtOAc:hexane = 5:5 v/v); colorless solid; mp 112 °C; IR (CHCl_3_) ** 3400–3100 (OOH), 1784, 1693 (C=O); ^1^H-NMR (CDCl_3_) *δ* 11.62 (1H, s, OOH) 7.37–7.27 (5H, m, arom H), 4.76 (1H, d, *J* = 14.7, CH_2_) 4.67 (1H, d, *J* = 14.7 Hz, CH_2_), 3.74 (1H, d, *J* = 17.7, CH_2_), 3.64 (1H, d, *J* = 17.4 Hz, CH_2_), 1.75 (2H, t, *J* = 8.4 Hz, CH_2_), 1.31 (2H, m, CH_2_), 0.88 (3H, t, *J* = 7.4 Hz, Me); ^13^C-NMR (CDCl_3_) *δ* 204.8 (C-4, C=O), 171.1 (C-2, C=O), 134.2, 129.1, 128.4, 128.3 (arom C), 85.7 (C-3), 54.1 (C-5, CH_2_), 46.8 (PhCH_2_), 33.5, 15.9 (CH_2_), 14.1 (Me). FAB HRMS (acetone/NBA) calcd for C_14_H_17_NO_4_Na 286.1055 (M+Na). Found 286.1032.

*1-Benzyl-3-butyl-3-hydroperoxypyrrolidine-2,4-dione* (**4d**).Yield (257.9 mg, 93%); *R*_f_ = 0.58 (EtOAc:hexane = 5:5 v/v); colorless solid; mp 80–81 °C; IR (CHCl_3_) ** 3400–3100 (OOH), 1784, 1695 (C=O); ^1^H-NMR (CDCl_3_) *δ* 11.37 (1H, s, OOH), 7.38–7.26 (5H, m, arom H), 4.72 (2H, s, CH_2_), 3.74 (1H, d, *J* = 17.6 Hz, CH_2_), 3.64 (1H, d, *J* = 17.6 Hz, CH_2_), 1.79 (2H, t, *J* = 8.1 Hz, CH_2_), 1.18 (4H, m, 2CH_2_), 0.84 (3H, t, *J* = 6.9 Hz, Me); ^13^C-NMR (CDCl_3_) *δ* 204.8 (C-4, C=O), 171.0 (C-2, C=O), 134.2, 129.1, 128.4, 128.3 (arom C), 85.7 (C-3), 54.1 (C-5, CH_2_), 46.8 (PhCH_2_), 31.4, 24.4, 22.8 (CH_2_), 13.6 (Me). FAB HRMS (acetone/NBA) calcd for C_15_H_19_NO_4_Na 300.1212 (M+Na). Found 300.1219.

*1-Benzyl-3-hexyl-3-hydroperoxypyrrolidine-2,4-dione* (**4e**).Yield (293.2 mg, 96%); *R*_f_ = 0.60 (EtOAc:hexane = 4: 6 v/v); colorless solid; mp 78–80 °C; IR (CHCl_3_) ** 3400–3100 (OOH), 1784, 1691 (C=O); ^1^H-NMR (CDCl_3_) *δ* 11.56 (1H, s, OOH), 7.38–7.27 (5H, m, arom H), 4.75 (1H, d,  *J* = 15.0 Hz, CH_2_), 4.69 (1H, d, *J* = 15.0 Hz, CH_2_), 3.75 (1H, d, *J* = 18.0 Hz, CH_2_), 3.64 (1H, d,  *J* = 18.0 Hz, CH_2_), 1.78 (2H, t, *J* = 7.8 Hz, CH_2_), 1.21 (8H, m, 4xCH_2_), 0.85 (3H, t, *J* = 6.5 Hz, Me); ^13^C-NMR (CDCl_3_) *δ* 204.8 (C-4, C=O), 171.1 (C-2, C=O), 134.2, 129.1, 128.4, 128.3 (arom C), 85.7 (C-3), 54.1 (C-5, CH_2_), 46.8 (PhCH_2_), 31.6, 31.3, 29.2, 22.4 (2C) (CH_2_), 14.0 (Me). FAB HRMS (acetone/NBA) calcd for C_17_H_23_NO_4_Na 328.1525 (M+Na). Found 328.1493.

*1-Butyl-3-hydroperoxy-3-methylpyrrolidine-2,4-dione* (**4f**).Yield (183.0 mg, 91%); *R*_f_ = 0.52 (EtOAc:hexane = 6:4 v/v); colorless liquid; IR (CHCl_3_) ** 3400–3100 (OOH), 1784, 1693 (C=O);  ^1^H-NMR (CDCl_3_) *δ* 11.75 (1H, s, OOH), 3.90 (2H, s, CH_2_), 3.53 (2H, t, *J* = 6.9 Hz, CH_2_), 1.60 (2H, m, CH_2_), 1.36 (2H, m, CH_2_), 1.35 (3H, s, CH_3_), 0.96 (3H, t, *J* = 7.2 Hz, Me); ^13^C-NMR (CDCl_3_) *δ* 204.9 (C-4, C=O), 171.3 (C-2, C=O), 82.2 (C-3), 53.9 (C-5, CH_2_), 42.6, 28.6, 19.9 (CH_2_), 16.6, 13.7 (Me). FAB HRMS (acetone/NBA) calcd for C_9_H_15_NO_4_Na 224.0899 (M+Na). Found 224.0897.

*1-Butyl-3-ethyl-3-hydroperoxypyrrolidine-2,4-dione* (**4g**).Yield (193.7 mg 90%); *R*_f_ = 0.66 (EtOAc:hexane = 6:4 v/v); colorless liquid; IR (CHCl_3_) ** 3400–3100 (OOH), 1782, 1689 (C=O);  ^1^H-NMR (CDCl_3_) *δ* 11.85 (1H, s, OOH), 3.90 (1H, d, *J* = 18.0 Hz, CH_2_), 3.83 (1H, d, *J* = 18.0 Hz, CH_2_), 3.63 (1H, m, CH_2_), 3.48 (1H, m, CH_2_), 1.79 (2H, q, *J* = 7.2 Hz, CH_2_), 1.61 (2H, m, CH_2_), 1.39 (2H, m, CH_2_), 0.96 (3H, t, *J* = 7.2 Hz, Me), 0.88 (3H, t, *J* = 7.5 Hz, Me); ^13^C-NMR (CDCl_3_) *δ* 205.3 (C-4, C=O), 170.7 (C-2, C=O), 85.7 (C-3), 54.5 (C-5, CH_2_), 42.6, 28.4, 24.6, 19.7 (CH_2_), 13.4, 6.7 (Me). FAB HRMS (acetone/NBA) calcd for C_10_H_17_NO_4_Na 238.1055 (M+Na). Found 238.1036.

*1,3-Dibutyl-3-hydroperoxypyrrolidine-2,4-dione* (**4h**).Yield (231.1 mg, 95%); *R*_f_ = 0.53 (EtOAc:hexane = 5:5 v/v); colorless liquid; IR (CHCl_3_) ** 3400–3100 (OOH), 1784, 1689 (C=O);  ^1^H-NMR (CDCl_3_) *δ* 11.85 (1H, s, OOH), 3.88 (1H, d, *J* = 18.0 Hz, CH_2_), 3.83 (1H, d, *J* = 18.0 Hz, CH_2_), 3.61 (1H, m, CH_2_), 3.48 (1H, m, CH_2_), 1.73 (2H, t, *J* = 7.8 Hz, CH_2_), 1.59 (2H, m, CH_2_), 1.37 (2H, m, CH_2_), 1.25 (4H, m, 2CH_2_), 0.96 (3H, t, *J* = 7.2 Hz, Me), 0.85 (3H, t, *J* = 6.8 Hz, Me);  ^13^C-NMR (CDCl_3_) *δ* 205.3 (C-4, C=O), 170.8 (C-2, C=O), 85.3 (C-3), 54.4 (C-5, CH_2_), 42.3, 30.9, 28.3, 24.2, 22.5, 19.6 (CH_2_), 13.4, 13.3 (Me). FAB HRMS (acetone/NBA) calcd for C_12_H_21_NO_4_Na 266.1368 (M+Na), Found 266.1375.

*1-(*t*-Butyl)-3-hydroperoxy-3-methylpyrrolidine-2,4-dione* (**4i**).Yield (181.0 mg, 90%); *R*_f_ = 0.55 (EtOAc:hexane = 7:3 v/v); colorless solid; mp 99–100 °C; IR (CHCl_3_) ** 3400–3100 (OOH), 1782, 1697 (C=O); ^1^H-NMR (CDCl_3_) *δ* 11.27 (1H, s, OOH), 3.94 (2H, s, CH_2_-C=O), 1.49 (9H, s, *t*-Bu), 1.32 (3H, s, Me); ^13^C-NMR (CDCl_3_) *δ* 205.3 (C-4, C=O), 171.5 (C-2, C=O), 83.2 (C-3), 55.8 (>C<), 52.8 (C-5, CH_2_), 28.1 (3CH_3_), 16.9 (Me). FAB HRMS (acetone/NBA) calcd for C_9_H_16_NO_4_ 202.1079 (M+H). Found 202.1069.

*1-(*t*-Butyl)-3-ethyl-3-hydroperoxypyrrolidine-2,4-dione* (**4j**).Yield (206.6 mg, 96%); *R*_f_ = 0.64 (EtOAc:hexane = 4:6 v/v); colorless solid; mp 130–131 °C; IR (CHCl_3_) ** 3500–3200 (OOH), 1780, 1695 (C=O); ^1^H-NMR (CDCl_3_) *δ* 12.34 (1H, s, OOH), 4.08 (1H, d, *J* = 18.3 Hz, CH_2_), 3.97 (1H, d,  *J* = 18.3 Hz, CH_2_), 1.58 (2H, q, *J* = 7.5 Hz, CH_2_), 1.41 (9H, s, *t*-Bu), 0.77 (3H, t, *J* = 7.5 Hz, Me);
^13^C-NMR (CDCl_3_) *δ* 207.7 (C-4, C=O), 169.2 (C-2, C=O), 85.3 (C-3), 54.5 (>C<), 53.3 (C-5, CH_2_), 27.1 (3Me), 24.6 (CH_2_), 16.9 (Me). FAB HRMS (acetone/NBA) calcd for C_10_H_17_NO_4_Na 238.1055 (M+Na). Found 238.1045.

*1-(*t*-Butyl)-3-hydroperoxy-3-propylpyrrolidine-2,4-dione* (**4k**).Yield (227.1 mg, 99%); *R*_f_ = 0.44 (EtOAc:hexane = 4:6 v/v); colorless solid; mp 112–113 °C; IR (CHCl_3_) ** 3450–3050 (OOH), 1782, 1685 (C=O); ^1^H-NMR (CDCl_3_) *δ* 11.59 (1H, s, OOH), 3.95 (1H, d, *J* = 17.8 Hz, CH_2_), 3.84 (1H, d,  *J* = 17.8 Hz, CH_2_), 1.69 (2H, t, *J* = 8.4 Hz, CH_2_), 1.49 (9H, s, *t*-Bu), 1.29 (2H, m, CH_2_), 0.88 (3H, t,  *J* = 7.1 Hz, Me); ^13^C-NMR (CDCl_3_) *δ* 205.7 (C-4, C=O), 171.3 (C-2, C=O), 86.5 (C-3), 55.8 (>C<), 53.3 (C-5, CH_2_), 33.7 (CH_2_), 27.5 (3Me), 15.9 (CH_2_), 14.1 (Me). FAB HRMS (acetone/NBA) calcd for C_11_H_20_NO_4_ 230.1392 (M+H). Found 230.1042.

*1-(*t*-Butyl)-3-butyl-3-hydroperoxypyrrolidine-2,4-dione* (**4l**).Yield (240.9 mg, 99%); *R*_f_ = 0.58 (EtOAc:hexane = 4:6 v/v); colorless solid; mp 87–88 °C; IR (CHCl_3_) ** 3400–3100 (OOH), 1782, 1685 (C=O); ^1^H-NMR (CDCl_3_) *δ* 11.47 (1H, s, OOH), 3.95 (H, d, *J* = 18.0 Hz, CH_2_), 3.83 (1H, d, *J* = 18.0 Hz, CH_2_), 1.72 (2H, t, *J* = 7.2 Hz, CH_2_), 1.49 (9H, s, *t*-Bu), 1.25 (4H, m, 2CH_2_), 0.82 (3H, t, *J* = 7.1 Hz, Me); ^13^C-NMR (CDCl_3_) *δ* 205.8 (C-4, C=O), 171.2 (C-2, C=O), 86.4 (C-3), 55.8 (>C<), 53.6 (C-5, CH_2_), 31.4 (CH_2_), 27.5 (3Me), 24.4, 22.7 (CH_2_), 13.5 (Me). FAB HRMS (acetone/NBA) calcd for C_12_H_22_NO_4_ 244.1549 (M+H). Found 244.1521.

*1-(t-Butyl)-3-hexyl-3-hydroperoxypyrrolidine-2,4-dione* (**4m**).Yield (276.9 mg, 98%); *R*_f_ = 0.45 (EtOAc:hexane = 3:7 v/v); colorless solid; mp 94 °C; IR (CHCl_3_) ** 3400–3100 (OOH), 1780, 1685 (C=O); ^1^H-NMR (CDCl_3_) *δ* 11.20 (1H, s, OOH), 3.94 (H, d, *J* = 17.4 Hz, CH_2_), 3.83 (1H, d, *J* = 17.4 Hz, CH_2_), 1.71 (2H, m, CH_2_), 1.49 (9H, s, *t*-Bu), 1.27 (8H, m, 4CH_2_), 0.85 (3H, t, *J* = 6.9 Hz, Me);  ^13^C-NMR (CDCl_3_) *δ* 205.7 (C-4, C=O), 171.3 (C-2, C=O), 86.6 (C-3), 55.9 (>C<), 53.6 (C-5, CH_2_), 31.8, 31.3, 29.2 (CH_2_), 27.6 (3Me), 22.4, 22.3 (CH_2_), 13.97 (Me). FAB HRMS (acetone/NBA) calcd for C_14_H_26_NO_4_ 272.1862 (M+H). Found 272.1857.

*3-Hydroperoxy-3-methyl-1-(*i*-propyl)pyrrolidine-2,4-dione* (**4n**).Yield (177.8 mg, 95%); *R*_f_ = 0.41 (EtOAc:hexane = 7:3 v/v); colorless solid; mp 82–84 °C; IR (CHCl_3_) ** 3400–3100 (OOH), 1784, 1685 (C=O); ^1^H-NMR (CDCl_3_) *δ* 11.65 (1H, s, OOH), 4.66 (1H, sep, *J* = 6.6 Hz, CH), 3.87 (1H, d, *J* = 17.7 Hz, CH_2_), 3.80 (1H, d, *J* = 17.7 Hz, CH_2_), 1.32 (3H, s, Me), 1.25 (3H, d, *J* = 6.6 Hz, Me), 1.22 (3H, d,  *J* = 6.6 Hz, Me); ^13^C-NMR (CDCl_3_) *δ* 204.9 (C-4, C=O), 170.5 (C-2, C=O), 82.6 (C-3), 49.0 (C-5, CH_2_), 43.1 (CH), 19.43, 18.88, 16.36 (Me). FAB HRMS (acetone/NBA) calcd for C_8_H_13_NO_4_Na 210.0742 (M+Na). Found 210.0740.

*3-Ethyl-3-hydroperoxy-1-(*i*-propyl)pyrrolidine-2,4-dione* (**4o**).Yield (195.2 mg, 97%); *R*_f_ = 0.50 (EtOAc:hexane = 7:3 v/v); colorless solid; mp 63–65 °C; IR (CHCl_3_) ** 3600–3100 (OOH), 1782, 1685 (C=O); ^1^H-NMR (CDCl_3_) *δ* 11.66 (1H, s, OOH), 4.69 (1H, sep, *J* = 6.6 Hz, CH), 3.85 (1H, d, *J* = 17.4 Hz, CH_2_), 3.71 (1H, d, *J* = 17.4 Hz, CH_2_), 1.79 (2H, q, *J* = 7.3 Hz, CH_2_), 1.26 (3H, d, *J* = 6.6 Hz, Me), 1.23 (3H, d, *J* = 6.6 Hz, Me), 0.78 (3H, t, *J* = 7.3 Hz, Me); ^13^C-NMR (CDCl_3_) *δ* 205.3 (C-4, C=O), 170.1 (C-2, C=O), 86.4 (C-3), 49.7 (C-5, CH_2_), 43.1 (CH), 24.7 (CH_2_), 19.7, 18.9, 6.8 (Me). FAB HRMS (acetone/NBA) calcd for C_9_H_15_NO_4_Na 224.0899 (M+Na). Found 224.0895.

*3-Hydroperoxy-1-(*i*-propyl)-3-propylpyrrolidine-2,4-dione* (**4p**).Yield (211.0 mg, 98%); *R*_f_ = 0.59 (EtOAc:hexane = 7:3 v/v); colorless solid; mp 78 °C; IR (CHCl_3_) ** 3600–3100 (OOH), 1782, 1685 (C=O); ^1^H-NMR (CDCl_3_) *δ* 11.71 (1H, s, OOH), 4.68 (1H, sep, *J* = 6.6 Hz, CH), 3.85 (1H, d, *J* = 17.7 Hz, CH_2_), 3.72 (1H, d, *J* = 17.7 Hz, CH_2_), 1.71 (2H, t, *J* = 8.4 Hz, CH_2_), 1.30 (2H, m, CH_2_), 1.26 (3H, d,  *J* = 6.6 Hz, Me), 1.22 (3H, d, *J* = 6.6 Hz, Me), 0.88 (3H, t, *J* = 7.2 Hz, Me); ^13^C-NMR (CDCl_3_) *δ* 205.3 (C-4, C=O), 170.2 (C-2, C=O), 86.0 (C-3), 49.7 (C-5, CH_2_), 43.0 (CH), 33.3 (CH_2_), 19.6, 18.9 (Me), 15.9 (CH_2_), 14.0 (Me). FAB HRMS (acetone/NBA) calcd for C_10_H_17_NO_4_Na 238.1055 (M+Na). Found 238.1044.

*5-Hydroperoxy-5-*i*-propryl-1,3-dimethylbarbituric acid*(**6a**). Colorless oil; IR (CHCl_3_) ** 3600–3100 (OOH), 1691 (C=O); ^1^H-NMR (300 MHz, CDCl_3_) *δ* 10.35 (1H, s, OOH), 3.37 (6H, s, 2 × CH_3_), 2.38 (1H, sep, *J* = 6.9 Hz, CH_3_-CH-CH_3_), 0.97 (6H, d, *J* = 6.9 Hz, CH_3_-CH-CH_3_); ^13^C-NMR (75 MHz, CDCl_3_) *δ* 168.1, 150.7 (C=O), 89.5 (>C-O), 36.9 (CH_3_-CH-CH_3_), 29.0 (CH_3_), 16.6 (CH_3_-CH-CH_3_). FAB HRMS (acetone/NBA) calcd for C_9_H_14_N_2_O_5_ 231.0903 (M+H). Found231.0982.

*5-Benzyl-5-hydroperoxy-1,3-dimethylbarbituric acid*(**6b**). Colorless blocks (from CH_2_Cl_2_/hexane); mp 130 °C; IR (KBr) ** 3500–3200 (OOH), 1697, 1678 (C=O); ^1^H-NMR (300 MHz, CDCl_3_) *δ* 10.67 (1H, s, OOH), 7.28-7.26 (3H, m, arom H), 7.00–6.96 (2H, m, arom H), 3.25 (2H, s, CH_2_), 3.13 (6H, s,
2 × CH_3_); ^13^C-NMR (75 MHz, CDCl_3_) *δ* 168.3, 149.4 (C=O), 130.2 (arom C), 129.1, 128.7, 128.6, 128.5 (arom CH), 87.2 (>C-O), 42.7 (CH_2_), 28.6 (CH_3_). Anal. Calcd for C_13_H_14_N_2_O_5_: C, 56.11; H, 5.07; N, 10.07. Found: C, 55.87; H, 5.01; N, 10.18.

*5-Hydroperoxy-5-[(4-methoxyphenyl)methyl]-1,3-dimethylbarbituric acid*(**6c**). Colorless blocks (from CH_2_Cl_2_/hexane); mp 117 °C; IR (KBr) ** 3600–3100 (OOH), 1728, 1692 (C=O); ^1^H-NMR (300 MHz, CDCl_3_) *δ* 10.85 (1H, s, OOH), 6.88-6.75 (4H, m, arom H), 3.76 (3H, s, CH_3_O-), 3.19 (2H, s, CH_2_), 3.16 (6H, s, 2 × CH_3_); ^13^C-NMR (75 MHz, CDCl_3_) *δ* 168.3, 159.5, 149.5 (C=O), 121.9 (arom C), 130.2, 114.0 (arom CH), 87.2 (>C-O), 55.1 (CH_3_O), 41.8 (-CH_2_-), 28.6 (CH_3_). Calcd for C_14_H_16_N_2_O_6_: C, 54.54; H, 5.23; N, 9.09. Found: C, 54.51; H, 5.21; N, 9.13.

*5-Hydroperoxy-5-[(2-methoxyphenyl)methyl]-1,3-dimethylbarbituric acid*(**6d**). Colorless plates (from CH_2_Cl_2_/hexane); mp 148–150 °C (decompd); IR (KBr) ** 3500–3200 (OOH), 1674 (C=O); ^1^H-NMR (300 MHz, CDCl_3_) *δ* 9.15 (1H, br, OOH), 7.27-6.78 (4H, m, arom H), 3.77 (3H, s, CH_3_O), 3.27 (2H, s, -CH_2_-), 3.14 (6H, s, 2 × CH_3_); ^13^C-NMR (75 MHz, CDCl_3_) *δ* 167.9, 157.3, 150.0 (C=O), 131.7, 129.9, 120.5, 110.2 (arom CH), 119.1 (arom C), 86.9 (>C-O), 55.3 (CH_3_O), 37.2 (-CH_2_-), 28.6 (CH_3_). Anal. Calcd for C_14_H_16_N_2_O_6_: C, 54.54; H, 5.23; N, 9.09. Found: C, 54.55; H, 5.15; N, 9.18.

*5-Hydroperoxy-5-(2-naphthyl)methyl-1,3-dimethylbarbituric acid*(**6e**). Colorless microcrystals (from CH_2_Cl_2_/hexane); mp 135 °C; IR (KBr) ** 3500–3200 (OOH), 1705, 1678 (C=O); ^1^H-NMR (300 MHz, CDCl_3_) *δ* 10.0 (1H, br, OOH), 7.80-7.03 (7H, m, arom H), 3.41 (2H, s, CH_2_), 3.08 (6H, s, 2 × CH_3_); ^13^C-NMR (75 MHz, CDCl_3_) *δ* 168.5, 149.4 (C=O), 133.2, 132.8, 127.6 (arom C), 128.7, 128.6, 127.8, 127.5, 126.9, 126.74, 126.4 (arom CH), 87.3 (>C-O), 42.9 (CH_2_), 28.7 (CH_3_). Anal. Calcd for C_17_H_16_N_2_O_5_: C, 62.19; H, 4.91; N, 8.53. Found: C, 62.45; H, 4.85; N, 8.54.

*3-Butyl-3-hydroperoxy-1*H*-quinoline-2,4-dione* (**8**). Pale yellow microcrystals (from EtOAc/hexane); mp 121 °C; IR (KBr) ** 3429 (OOH), 3350–3100 (NH), 1720, 1693, 1666 (C=O); ^1^H-NMR (300 MHz, DMSO-*d*_6_) *δ* 12.2 (1H, s, OOH), 11.0 (1, s, NH), 7.80–7.56 (2H, m, arom H), 7.14–7.03 (2H, m, arom H), 1.78–1.73 (2H, m, CH_2_), 1.14–1.05 (4H, m, -CH_2_-CH_2_-CH_2_-CH_3_), 0.72 (3H, t, *J* = 6.9 Hz, -CH_3_); ^13^C-NMR (75 MHz, CDCl_3_) *δ* 193.9, 170.1 (C=O), 141.9, 119.0 (arom C), 136.8, 126.6, 122.8, 116.6 (arom CH), 88.6 (>C-O), 35.3 (-CH_2_-CH_2_-CH_2_-CH_3_), 24.2 (-CH_2_-CH_2_-CH_2_-CH_3_), 22.1 (-CH_2_-CH_2_-CH_2_-CH_3_), 13.4 (-CH_2_-CH_2_-CH_2_-CH_3_). FAB HRMS (acetone/NBA) calcd for C_13_H_16_NO_4_ 250.1079 (M+H). Found250.1062.

The physical data for the alcohols **9a**–**d**, **9g**, and **10b** are given below.

*4-Hydroxy-4-methyl-1,2-diphenylpyrazolidine-3,5-dione* (**9a**). Colorless microcrystals (from CH_2_Cl_2_/hexane); mp 172–173 °C; IR (KBr) ** 35100–3100 (OH), 1757, 1722 (C=O); ^1^H-NMR (300MHz, CDCl_3_) *δ* 7.33–7.17 (10H, m, arom H), 4.23 (1H, s, OH), 1.71 (3H, s, CH_3_); ^13^C-NMR (75 MHz, CDCl_3_) *δ* 170.8 (C=O), 135.1 (arom C), 129.0, 127.2, 122.7 (arom CH), 71.1 (>C-O), 23.4 (CH_3_). Anal. Calcd for C_16_H_14_N_2_O_3_: C, 68.07; H, 5.00; N, 9.92. Found: C, 68.05; H, 4.97; N, 9.96.

*4-Ethyl-4-Hydroxy-1,2-diphenylpyrazolidine-3,5-dione* (**9b**). IR (KBr) ** 3500–3150 (OH), 1759, 1720 (C=O); ^1^H-NMR (300 MHz, CDCl_3_) *δ* 7.50-7.00 (10H, m, arom H), 4.54 (1H, s, OH), 2.05 (2H, q,  *J* = 7.2 Hz, -CH_2_-CH_3_), 0.95 (3H, t, *J* = 7.2 Hz, -CH_2_-CH_3_); ^13^C-NMR (75 MHz, CDCl_3_) *δ* 170.6 (C=O), 134.7 (arom C), 128.9, 127.1, 122.8 (arom CH), 74.7 (>C-O), 30.6 (-CH_2_-CH_3_, Et), 7.0 (-CH_2_-CH_3_, Et).

*4-Hydroxy-4-propyl-1,2-diphenylpyrazolidine-3,5-dione* (**9c**). Colorless blocks (from Et_2_O/hexane); mp 160 °C (lit, mp 167–169 °C); IR (KBr) ** 3500–3200 (OH), 1759, 1724 (C=O); ^1^H-NMR (300 MHz, CDCl_3_) *δ* 7.33–7.16 (10H, m, arom H), 3.73 (1H, s, OH), 2.06–2.00 (2H, m, -CH_2_-C_2_H_5_), 1.48–1.40 (2H, m, -CH_2_-CH_2_-CH_3_), 0.94 (3H, t, *J* = 7.2 Hz, -CH_2_-CH_2_-CH_3_); ^13^C-NMR (75 MHz, CDCl_3_) *δ* 170.3 (C=O), 135.1 (arom C), 129.0, 127.2, 122.7 (arom CH), 74.0 (>C-O), 39.7 (-CH_2_-CH_2_-CH_3_), 16.1 (-CH_2_-CH_2_-CH_3_), 13.9 (-CH_2_-CH_2_-CH_3_). Anal. Calcd for C_18_H_18_N_2_O_3_: C, 69.66; H, 5.85; N, 9.03. Found: C, 69.71; H, 5.81; N, 8.86.

*4-Hydroxy-4-*i*-propyl-1,2-diphenylpyrazolidine-3,5-dione* (**9d**). Pale yellow plates (from Et_2_O/hexane); mp 175 °C (decompd); IR (KBr) ** 3550–3000 (OH), 1759, 1720 (C=O); ^1^H-NMR (300 MHz, CDCl_3_) *δ* 7.32–7.14 (10H, m, arom H), 3.85 (1H, br. S, OH), 2.34 (1H, sep, *J* = 6.9 Hz, CH_3_-CH-CH_3_), 1.15 (6H, d, *J* = 6.9 Hz, CH_3_-CH-CH_3_); ^13^C-NMR (75 MHz, CDCl_3_) *δ* 17.1 (C=O), 134.8 (arom C), 129.0, 127.0, 122.7 (arom CH), 76.3 (>C-O), 36.2 (CH_3_-CH-CH_3_), 15.6 (CH_3_-CH-CH_3_). Anal. Calcd for C_18_H_18_N_2_O_3_: C, 69.66; H, 5.85; N, 9.03. Found: C, 69.91; H, 5.50; N, 9.01.

*4-Benzyl-4-hydroxy-1,2-diphenylpyrazolidine-3,5-dione* (**9g**). Colorless needles (from EtOAc/hexane); mp 200–210 °C (decompd) (lit, mp 206–208 °C); IR (KBr) ** 3500–3100 (OH), 1764, 1705 (C=O);  ^1^H-NMR (300 MHz, CDCl_3_) *δ* 7.25-6.75 (15H, m, arom H), 4.88 (1H, s, OH), 3.44 (2H, s, CH_2_);  ^13^C-NMR (75 MHz, CDCl_3_) *δ* 169.2 (C=O), 133.8, 131.6, 130.2 (arom C), 128.7, 128.6, 127.6, 127.2, 123.4 (arom CH), 75.8 (>C-O), 43.7 (CH_2_). Anal. Calcd for C_22_H_18_N_2_O_3_: C, 73.73; H, 5.06; N, 7.82. Found: C, 73.47; H, 4.82; N, 7.76.

*5-Benzyl-5-hydroxy-1,3-dimethylbarbituric acid*(**10b**). Colorless blocks (from CH_2_Cl_2_/hexane); mp 112 °C; IR (KBr) ** 3500–3200 (OH), 1700, 1681 (C=O); ^1^H-NMR (300 MHz, CDCl_3_) *δ* 7.29–7.26 (3H, m, arom H), 6.98–6.95 (2H, m, arom H), 4.11 (1H, br, OH), 3.28 (2H, s, CH_2_), 3.10 (6H, s, 2 × CH_3_); ^13^C-NMR (75 MHz, CDCl_3_) *δ* 169.9, 149.7 (C=O), 131.8 (arom C), 129.1, 128.5, 128.4 (arom CH), 76.6 (>C-O), 49.5 (CH_2_), 28.4 (CH_3_). Anal. Calcd for C_13_H_14_N_2_O_4_: C, 59.54; H, 5.38; N, 10.68. Found: C, 59.30; H, 5.33; N, 10.68.

The physical data for the alcohols **12a** and **12b** are given below.

*2-Benzyl-2-hydroxy-*N^1^,N^3^*-dipropylmalonamide* (**12a**). Yield (18%); *R*_f_ = 0.79 (Et_2_O); orange microcrystals; mp 74–76 °C; IR (KBr) ** 3398, 3360, 3312 (OH and NH), 1653 (C=O); ^1^H-NMR
(300 MHz, CDCl_3_) *δ* 7.40 (2H, br. s), 7.27-7.23 (5H, m), 4.88 (1H, br. s), 3.21–3.15 (6H, m), 1.49 (4H, sex, *J* = 7.3 Hz), 0.85 (6H, t, *J* = 7.3 Hz); ^13^C-NMR (75 MHz, CDCl_3_)* * 170.9, 134.9, 130.3, 128.0, 127.0, 77.8, 46.0, 41.5, 22.5, 11.3. Anal. Calcd for C_16_H_24_N_2_O_3_: C, 65.73; H, 8.27; N, 9.58. Found: C, 65.43; H, 8.56; N, 9.37.

*2-Benzyl-*N^1^,N^3^*-dibutyl-2-hydroxymalonamide* (**12b**). Yield (25%); *R*_f_ = 0.79 (Et_2_O); orange microcrystals; mp 75–77 °C; IR (KBr) ** 3377, 3314 (NH and OH), 1651 (C=O); ^1^H-NMR (300 MHz, CDCl_3_) *δ* 7.36 (2H, br.s), 7.26–7.22 (5H, m), 4.86 (1H, br. s), 3.23 (4H, q, *J* = 6.4 Hz), 3.14 (2H, s), 1.43 (2H, quin, *J* = 7.3 Hz), 1.27 (2H, sex, *J* = 7.3 Hz), 0.89 (3H, t, *J* = 7.3 Hz); ^13^C-NMR (75 MHz, CDCl_3_) *δ* 170.9, 134.9, 130.3, 128.0, 127.0, 77.8, 46.0, 39.6, 31.3, 19.9, 13.7. Anal. Calcd for C_18_H_28_N_2_O_3_: C, 67.47; H, 8.81; N, 8.74. Found: C, 67.40; H, 8.96; N, 8.73. FAB HRMS (acetone/NBA) calcd for C_18_H_29_N_2_O_3_ 321.2178 (M+H). Found 321.2184.

The physical data for the alcohols **14a** and **14b** are given below.

*Methyl 3-hydroxy-2-oxotetrahydrofuran-3-carboxylate*(**14a**). Yellow oil; IR (KBr) ** 3600–3100 (OH), 1768, 1720 (C=O); ^1^H-NMR (300 MHz, CDCl_3_) *δ* 4.86 (1H, br, OH), 4.54–4.39 (2H, m, -CH_2_-), 3.83–3.79 (1H, m, -CH_2_-), 2.73–2.65 (1H, m, -CH_2_-), 2.36 (3H, s, Me); ^13^C-NMR (75 MHz, CDCl_3_) *δ* 205.7, 174.8 (C=O), 81.2 (>C-O), 66.0 (-CH_2_-), 33.8 (-CH_2_-), 24.5 (CH_3_).

*Ethyl 2-hydroxycyclopentanone-2-carboxylate*(**14b**). Pale yellow oil; IR (KBr) ** 3600–3100 (OH), 1757, 1732 (C=O); ^1^H-NMR (300 MHz, CDCl_3_) *δ* 4.28-4.21 (2H, m, -CH_2_-), 4.10 (1H, br, OH),
2.48–2.41 (3H, m, -CH_2_-), 2.13–2.07 (3H, m, -CH_2_-), 1.31–1.25 (3H, m, Me); ^13^C-NMR (75 MHz, CDCl_3_) *δ* 213.4, 171.5 (C=O), 79.7 (>C-O), 62.4 (-CH_2_-), 35.7 (-CH_2_-), 34.7 (-CH_2_-), 18.2 (-CH_2_-), 13.8 (CH_3_).
